# Practical Advice on Scientific Design of Freeze-Drying Process: 2023 Update

**DOI:** 10.1007/s11095-023-03607-9

**Published:** 2023-10-02

**Authors:** Serguei Tchessalov, Vito Maglio, Petr Kazarin, Alina Alexeenko, Bakul Bhatnagar, Ekneet Sahni, Evgenyi Shalaev

**Affiliations:** 1 Pfizer Inc, Andover, MA USA; 2 Birck Nanotechnology Center, Purdue University, 1205 W State St., West Lafayette, IN 47907 USA; 3 Regeneron, Troy, NY USA; 4 Pharmaceutical Sciences, R&D, Abbvie, Irvine, CA USA

**Keywords:** freeze-drying, lyophilization, mathematical modeling, process design

## Abstract

**Objective:**

The purpose of this paper is to re-visit the design of three steps in the freeze-drying process, namely freezing, primary drying, and secondary drying steps. Specifically, up-to-date recommendations for selecting freeze-drying conditions are provided based on the physical–chemical properties of formulations and engineering considerations.

**Methods and Results:**

This paper discusses the fundamental factors to consider when selecting freezing, primary drying, and secondary drying conditions, and offers mathematical models for predicting the duration of each segment and product temperature during primary drying. Three simple heat/mass transfer primary drying (PD) models were tested, and their ability to predict product temperature and sublimation time showed good agreement. The PD models were validated based on the experimental data and utilized to tabulate the primary drying conditions for common pharmaceutical formulations, including amorphous and partially crystalline products. Examples of calculated drying cycles, including all steps, for typical amorphous and crystalline formulations are provided.

**Conclusions:**

The authors revisited advice from a seminal paper by Tang and Pikal (Pharm Res. 21(2):191-200, 2004) on selecting freeze-drying process conditions and found that the majority of recommendations are still applicable today. There have been a number of advancements, including methods to promote ice nucleation and computer modeling for all steps of freeze-drying process. The authors created a database for primary drying and provided examples of complete freeze-drying cycles design. The paper may supplement the knowledge of scientists and formulators and serve as a user-friendly tool for quickly estimating the design space.

**Supplementary Information:**

The online version contains supplementary material available at 10.1007/s11095-023-03607-9.

## Introduction

Freeze-drying (lyophilization) is a common pharmaceutical manufacturing process used to produce various drug products. Freeze-drying consists of 3 segments, i.e., freezing, primary drying (ice sublimation), and secondary drying (desorption of unfrozen water), with primary drying (ice sublimation) being the longest. Freezing is a critical stage of a freeze-drying cycle, as the structure and morphology of a frozen cake can influence product behavior during primary drying. Freezing conditions could also majorly impact freeze-dried products' critical quality attributes and shelf life. The primary drying segment typically attracts the most attention because it provides the best opportunity to significantly reduce overall cycle time, while it may also be associated with significant product defects if performed under the wrong conditions.

Furthermore, product behavior during primary drying can be controlled by adjusting heat/mass transfer conditions and is far more predictable than freezing, which depends on a stochastic nucleation process. Acceleration of primary drying requires maintaining a higher product temperature, while product temperature should not exceed a critical product temperature limit to achieve a quality product. Secondary drying, during which an unfrozen fraction of water is removed from the product to achieve a target residual water content level, is usually a relatively straightforward part of freeze-drying.

In a seminal publication by Tang and Pikal[1], guidance on the selection of freeze-drying conditions was provided, along with a brief description of scientific principles behind the practical advice. It has been 18 years since the publication of this milestone paper by two co-authors from the University of Connecticut. The majority of recommendations from that paper are as relevant today as they were in 2004, and several exact citations from Tang and Pikal[1] paper are included in this manuscript. At the same time, there have been significant advancements in science and technology of freeze-drying, which warrant re-visiting. Freezing remains the most elusive part of freeze-drying, partly because of the fundamental unpredictability of a nucleation phenomenon. There are ongoing efforts to evaluate the feasibility of controlling ice nucleation at different scales. Studies have been performed to explore relationships between ice nucleation conditions and the quality of the finished product. As discussed in this paper, a mathematical model has also been developed to predict the freezing time. A significant part of this paper is focused (predictably) on primary drying, emphasizing building a design space for different products to provide initial guidance on process design for a laboratory freeze-dryer. For a scientist/formulator with limited freeze-drying experience, the database provides specific primary drying conditions for a range of formulations, vials, and fill volumes, while it can also be helpful for an advanced user. For example, the database provides a user-friendly way to explore design space. In a brief discussion of secondary drying, a simple computational model is introduced, which allows the estimation of secondary drying duration to achieve target residual water content for a typical amorphous product at different shelf temperatures. Suggestions on selecting conditions for all three stages of the freeze-drying process are provided, including examples of freeze-drying cycle recipes for amorphous and crystalline formulations.

## Selection of The Freeze-Drying Conditions: Overview

### Loading and Freezing

#### Overview

As “the theater begins with the cloakroom” (as attributed to a famous Russian art director Konstantin Stanislavsky), any freeze-drying recipe begins with the loading temperature. Freeze-dryers are usually loaded at either room temperature (usually 20 to 25°C) or 5°C when liquid stability is a potential concern. On rare occasions, a product is loaded onto shelves cooled below 0°C (e.g., to -50°C); this could be a case when a fast cooling rate is required if there are major concerns with liquid stability or for scheduling reasons in a manufacturing environment to reduce cycle time by eliminating a long cooling step. In such cases, water from the atmosphere could condense on the shelves to form ice, which is highly undesirable.

**Fig. 1:**
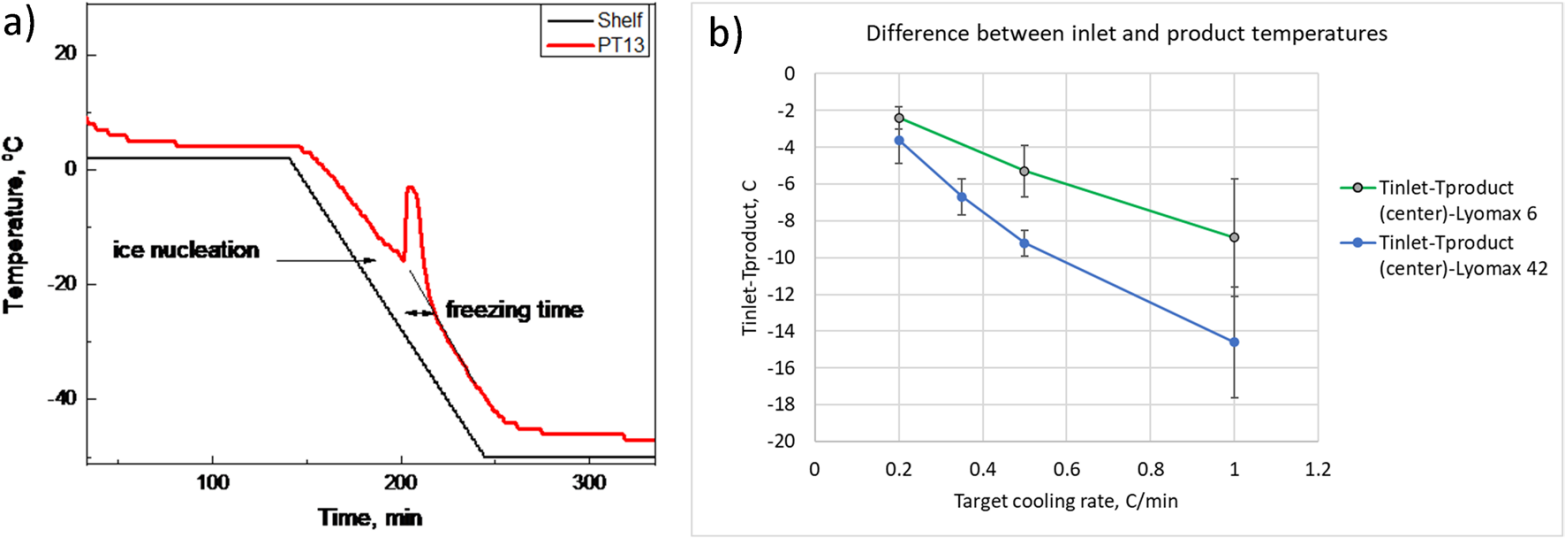
An example of product and shelf temperatures during cooling of a product in a lyophilizer (**a**). (**b**) represents an average difference between inlet (shelf) temperature and product temperature as a function of cooling rate for the pilot (green line) and commercial dryer (blue line).

After loading a freeze-dryer, vials are typically equilibrated for at least 30 min before cooling to minimize vial-to-vial temperature variations. In selecting the shelf cooling rate, one should remember that the shelf temperature/time program is not the same as the product temperature/time response, as illustrated in Fig. 1a. A faster cooling rate results in a larger difference between shelf and product temperature (Fig. 1b). In this example the larger dryer (Lyomax 42 with 42 sq.m shelf area) shows a more significant difference between inlet temperature and product temperature even when it was only partially loaded (2 shelves out of 15) as opposed to a fully loaded pilot dryer (6 sq.m). As discussed below, an approach to a cooling program depends on formulation type, i.e., if the formulation is completely amorphous or partially crystalline.

As illustrated in Fig. 1, product behavior during cooling can be described by two factors, i.e., ice nucleation temperature and freezing time. Ice nucleation can be measured directly by detecting an abrupt increase in the product temperature due to an exothermic process of ice formation. An exact definition of freezing time would require monitoring water-to-ice conversion in real-time, which would be a difficult task in practice. As a conceptual (and approximate) definition, freezing time can be defined as the period between ice nucleation and the time when the product temperature trend resumes following the shelf temperature trend. At this point, most “freezable” (in a kinetic sense) water is converted to ice, although water-to-ice transformation could probably continue below this point, depending on formulation composition and specifics of heat/mass transfer in a particular container. Operationally, the freezing time is commonly defined as the hold time at the final freezing temperature, sometimes including freezing ramp duration.

Lower supercooling (i.e., higher ice nucleation temperature) is considered beneficial, as it is expected to result in larger ice crystals with lower surface area [2, 3]. Frozen mass with larger and inter-connected ice crystals would have a lower resistance to water vapor transfer and a lower risk for protein destabilization because of a lower ice/solution interface and, therefore, a lower fraction of protein molecules exposure to the interface. A temperature equilibration step prior to the initiation of cooling is recommended [1] to reduce the vial-to-vial difference in ice nucleation. There is no definite agreement on the relationships between cooling rate and ice nucleation temperature. While it has been suggested that slow cooling causes larger supercooling [4], and it also reported that the cooling rate (range 0.6–40 K/min) did have some impact on the homogeneous ice nucleation temperature in solutions of LiCl with a concentration above 6.8 mol % [5], there are also reports to the contrary. Indeed, no impact of the cooling rate on the homogeneous ice nucleation temperature was observed for LiCl solutions at 5 mol % [5].

Furthermore, it was reported that an increase in the cooling rate from 0.1 to 1000 K/min resulted in only a 2°​C difference in the heterogeneous ice nucleation temperature and 4°​C in homogeneous ice nucleation [6]. No difference in the ice nucleation temperature was observed with cooling rates of 0.5 to 3.2°​C/min [7], 0.07-​7°​/min [8], and 0.05 to 1°​C/min [2] Also, nucleation rate coefficients (nucleation events per unit time per unit area) of ice on kaolinite did not demonstrate any significant difference in cooling rates between 0.8 and 10 K/min [9].

As noted in [1], “*it is not practical to manipulate the supercooling by changing the cooling rate in a freeze dryer because the cooling rates are usually limited to less than 2°C/min, and the degree of supercooling is unlikely to change within such a small range*.” It remains a true statement based on almost two decades since the publication of that paper.

One approach to eliminating the differences in freezing due to varying degrees of supercooling across a shelf of vials is by employing controlled ice nucleation. The product temperature is reduced below the equilibrium freezing point (or melting temperature). After a brief equilibration, ice nucleation is initiated by a variety of approaches: (i) pressurization and depressurization of the drying chamber [10]; (ii) introduction of an ice-fog [3]; (iii) reduction of chamber pressure [11]; and (iv) utilization of ultrasound [12, 13]. In addition, vacuum-induced surface freezing has also been explored [11, 14].

The use of higher ice nucleation temperatures results in the formation of larger ice crystals, which lead to larger pores post-ice sublimation and a lower cake resistance during drying. Consequently, primary drying duration can be reduced along with decreased inter-vial heterogeneity in drying rates [10, 15]**.**

Controlled ice nucleation (CIN) could be beneficial for amorphous formulations as it is associated with a lower dry layer resistance and less variability between vials [10, 15]. However, controlled ice nucleation may need longer/higher temperature secondary drying because of the lower specific surface area of the freeze-dried solids [16]. Solute crystallization may not always benefit from controlled ice nucleation, as observed in [17] using model water-NaCl mixtures. Controlled ice nucleation enabled control of the physical form of mannitol during freezing and facilitated the formation of anhydrous mannitol instead of the less desirable mannitol hemihydrate [18]. Implementing controlled ice nucleation in manufacturing is currently limited by the availability of freeze-dryers equipped with ice nucleation capabilities. Evaluating this technology at a laboratory scale is advisable as an alternative to annealing. Different methods of CIN were utilized to compare dried product attributes when nucleated at the same temperature [19–22]. Gitter et al.[19] conducted ice nucleation was achieved at -5°C using ice fog or depressurization methods in systems containing excipients (sucrose, trehalose) or their mixtures (mannitol and sucrose at a 4:1 ratio) and in monoclonal antibodies containing formulation (in sucrose, or trehalose, or mannitol-sucrose and 10 mM histidine buffer and 0.02% w/v polysorbate 80). The authors observed comparable product quality attributes covering a specific surface area, water content, phase behavior, turbidity post reconstitution, sub-visible particle counts, and % monomer / % High molecular weight (HMW) species (for the mAb containing formulation only). Vollrath et al. extended the earlier work to focus on ice fog methods only, namely, using the VERISEQ® nucleation system from IMA Life / Linder, the FreezeBooster® method (Millrock Technology) and the method described by Geidobler and coworkers [15]. Differences exist in the three methods in the temperature at which the ice is generated and the induction pressure, which could lead to the nucleation seeds of different ice morphologies (dendritic, hexagonal, and dispersed spherulitic) and, thereby, may later impact the ice fog texture. Ice nucleation was conducted at either -3°C or -10°C in monoclonal antibody formulations (10 mg/mL) in either sucrose or trehalose (disaccharide at 7.5% w/v). The authors observed a correlation between specific surface area (SSA) and ice nucleation temperature for the FreezeBooster® and Geidobler et al. methods for the sucrose-based formulations. No differences were observed in the SSAs of the trehalose-based formulations nucleated at -3(C or -10(C), suggesting that the disaccharide formulation can impact SSA, leading to differences in SSA between sucrose vs. trehalose-based formulations. The authors reported that the benefit of utilizing a higher ice nucleation temperature from the perspective of solid-state attributes could outweigh the higher risk of less complete nucleation. Luoma and coworkers investigated the effect of depressurization, partial vacuum, and ice fog techniques on product attributes of a monoclonal antibody formulation (10 and 100 mg/mL mAb in sucrose and histidine buffer-based formulations) and also an enzyme formulation (2.5 mg/mL in 500 mM arginine phosphate and polysorbate 80) post drying and during storage in vials of different sizes. Comparable product attributes were observed across the multiple mAb and enzyme formulations and vial sizes using different ice nucleation methods. The enzyme formulation cakes produced using the partial vacuum method were the exception to the general comparable behavior.

Freezing time is important because the material might be partially frozen at this stage, with the freeze-concentrated fraction containing protein in a liquid state. Protein molecules are susceptible to various stresses during this period, including, e.g., freeze-concentration, pH and ionic strength changes, ice/freeze concentrate interface, local mechanical stresses, and protein/cryoprotector phase separation. The freezing time may be reduced by using higher cooling rates, therefore reducing the risk of protein destabilization due to freezing stresses, but one needs to pay attention to a potential increase in heterogeneity of product properties.

Considering that a relatively fast cooling rate would probably have a minimal impact on supercooling and a potential benefit for reducing cycle time, a faster shelf cooling rate can be a logical recommendation for amorphous formulations. In the author's experience, an increase in cooling rate from 0.3 to 1°C/min resulted in a notable improvement of cake appearance for one of the small molecule drugs due to a reduction in phase separation. However, this advice may not apply to formulations with a crystalline bulking agent, as discussed below. The effect of fast cooling has been extensively studied in mannitol solutions, where vial breakage was reported post fast cooling and warming and attributed to mannitol + ice crystallization [23–25]. In addition, vial breakage during freezing might be a risk even for amorphous formulations if a faster cooling rate is used (unpublished data).

As for commercial cycles, cooling rates of 0.5°C/min or lower are recommended to ensure product temperature homogeneity across freeze-dryer shelves [26].

#### Annealing

1. Amorphous formulations
  In many cases, annealing is applied as a part of the freezing segment. As [1] wrote: “*Annealing above the glass transition temperature … causes growth of ice crystals, which decreases the product resistance to flow of water vapor and results in shorter primary drying time… the product specific surface area is reduced, which decreases the water desorption rate in secondary drying and may lead to increased residual moisture content in the final product or demand longer secondary drying*”.
  This statement remains valid for the amorphous formulations, clarifying that while annealing may shorten the duration of the primary drying, the overall cycle might still be longer because of the added annealing step. There are also observations that annealing can induce phase separation and skin formation on a frozen cake surface. Annealing increased drying duration due to increased dry layer resistance to mass transfer after more efficient excipient crystallization [27]. Given all positive and negative factors, we recommend testing the annealing approach in the product development phase. Annealing temperatures between -15°C and -10°C for 3–5 h is recommended as a temperature range above Tg’ for most of the products that use lyophilization. Also, the product temperature (including edge vials) during annealing should be lower than the melting temperature of the formulation. If product Tg’ is relatively high (e.g., -7 to -15°C), annealing may not be beneficial. In addition, for aggressive cycles with product T ~  > T_collapse_, Ostwald ripening of ice crystals can occur during primary drying because of a relatively high product temperature. In such cases, having an additional annealing step may not provide significant benefits in terms of cycle time reduction. It should also be noted that different annealing temperatures can influence the crystallization behavior of the excipients used in the formulation. This can potentially result in the formation of different polymorphs or hydrates, including the hemi-hydrate form of mannitol at warmer annealing temperatures. Therefore, the selection of the annealing temperature should also take into account the desired polymorphic form of the excipients. According to [18, 28], “ice crystallization followed by annealing at temperatures >  = 10 C can be an effective strategy to prevent mannitol hemi-hydrate formation.
2. Partially crystalline formulations
  For formulations with crystallizable solutes, there is an additional critical consideration, that is, solute + water secondary crystallization.^1^ Solute crystallization is highly desirable for crystalline bulking agents, mannitol, and glycine, whereas crystallization of buffer and lyoprotector should be avoided. A detrimental impact of crystallization of a lyoprotector during freeze-drying on protein activity has been described in [30]. In addition, the crystallization of PEG in PEGylated proteins was shown to affect protein stability negatively [31].
  The timing of an excipient crystallization can be important in some cases. It has been suggested, for example, that the crystallization of a bulking agent during cooling can be beneficial in minimizing vial breakage. A case study that describes vial breakage as the result of annealing, and prevention of the vial breakage using step-wise cooling, is provided [32]. Also, the impact of timing of crystallization of the bulking agent (e.g., if it takes place during cooling or at the beginning of primary drying) during freeze-drying on protein stability was reported in [33]; in that case, crystallization of solute during cooling was beneficial for stability. Results from these studies indicate the potential advantage of promoting the crystallization of a solute during cooling (rather than during annealing). There are two practical ways to facilitate such crystallization, i.e., by using a cooling rate lower than the critical cooling rate or applying step-wise cooling. In this context, the critical cooling rate is defined as the maximal cooling rate at which secondary solute + water crystallization (see comment on eutectic crystallization) occurs during cooling. Specifically, the solute would crystallize during cooling if the cooling rate is lower than the critical cooling rate; otherwise, if the cooling rate is higher than the critical cooling rate, the solute remains amorphous during cooling, although it may crystallize during subsequent heating or annealing.^2^ The critical cooling rate is predominantly determined by the composition of the solution, while other factors, such as the volume of the solution and heterogeneous surfaces, could also influence it. Critical cooling rates were determined for several model systems. In the glycine-sucrose-water system, for example, the critical cooling rate decreased from > 20°C/min for a solution with glycine/sucrose ratio of 3.0 to < 0.5°C/min at glycine/sucrose ratio of 0.9 [34]. For 5% mannitol solution in glass vials, the critical cooling rate was estimated to be between 0.2°C/min and 0.5°C/min [35]**.** While the lower cooling rate can play a role in initiating the crystallization of a solute during the cooling phase, it is important to note that complete crystallization often requires further treatment, such as annealing, particularly when only partial crystallization occurs during cooling. Furthermore, the ease of crystallization can be significantly impacted by the concentrations of the components within the formulation.
  From a practical perspective, when selecting set points for the freezing segment of a freeze-drying cycle for a formulation containing a crystallizable solute, it is necessary to choose a temperature and dwell time for a stepwise cooling program. These parameters should ideally lie between the equilibrium secondary melting temperature (e.g., mannitol + ice melting) and the glass transition temperature (Tg’), and can be guided by DSC and/or low-temperature XRD experiments. However, these are initial recommendations that should be fine-tuned based on experimental observations for each specific formulation. The question regarding hold time is less straightforward due to the limited research on the kinetics of solute crystallization in vials during freeze-drying [36, 37]. It is proposed to approach this question similarly to determining the final hold time, but again, this should be experimentally confirmed and adapted as necessary.

#### Final Freezing Temperature and Hold Time

Regarding final freezing temperature and hold time, Tang and Pikal[1] recommended targeting a final freezing product temperature of -40°C in most cases. This considers that if the *T*g’ (for completely amorphous products) or temperature of the secondary crystallization (amorphous/crystalline formulations) is usually higher than − 38°. Due to the radiation effect, shelf temperature should be lower (-45°C or lower) to ensure that product temperature in edge vials remains below Tg’. Tang and Pikal [1] recommended one-hour hold time at the final freezing temperature for products with fill depth <  = 1 cm, and two hours for a 1–2 cm fill depth. Fill heights above 2 cm are undesirable, but certain practical applications may require higher fill depth; in such cases, the freezing hold time should be extended beyond two hours. To assess freezing time, a simplified freezing model can be used [38]. The freezing model is based on the assumption of 0D transient heat conduction with lumped capacitance heat transfer. The lumped capacitance approach assumes that the internal resistance to thermal conduction within the product is much lower than the heat transfer between the product and its environment, resulting in a uniform temperature profile throughout the product. The error associated with lumped capacitance method can be characterized by the Biot number (Bi = hL/k, where h – heat transfer coefficient, L – characteristic length, which can be fill height of the product or vial diameter,k – product thermal conductivity). For low Biot numbers (typically Bi < 0.1), the temperature gradient in the product is small [39]**,** and inaccuracy linked to lumped capacitance method is low. In addition, it is assumed that the product temperature is constant during crystallization, and heat is supplied to the product from the shelf only. The lumped capacitance model agrees well with the experimental data [38]. The model requires several input parameters: fill volume, heat transfer coefficient, nucleation, and freezing temperatures. The stochastic nature of the nucleation can introduce uncertainty to the model predictions. The input parameters are to be obtained from the experimental data, or a typical value should be assigned. Use of controlled ice nucleation would reduce the parametric variability [10, 15].

**Fig. 2:**
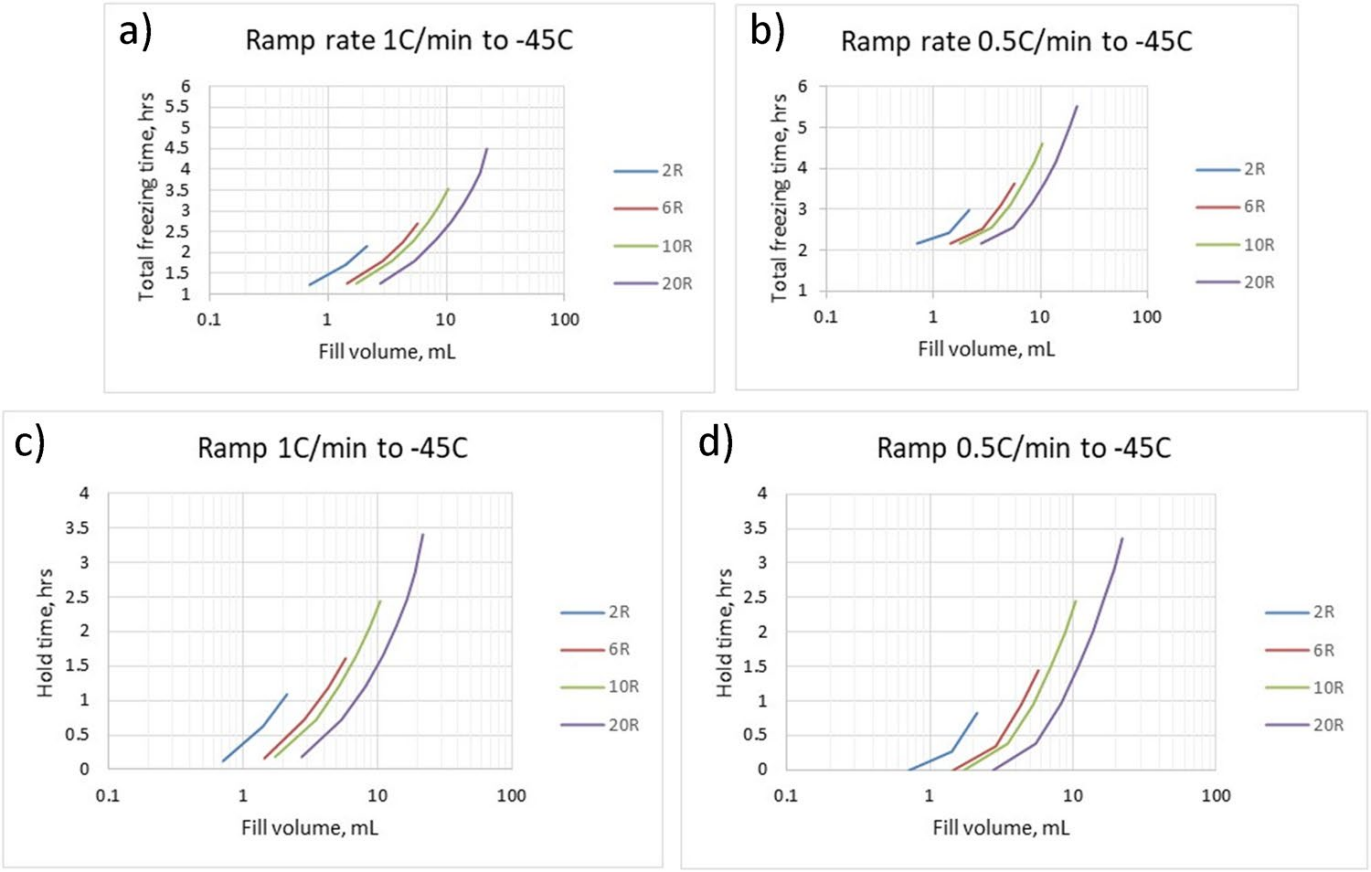
Freezing time to reach -44.8 °C is calculated using the freezing model [38]**.** The top panels (**a**,**b**) represent a total freezing time for the product (including the ramp) to reach -44.8°C at a cooling rate of 1°C/min (left panel) and 0.5°C/min (right panel) as a function of fill volume for different vial sizes. The bottom panels (**c**, **d**) are hold times (excluding ramp times) for similar scenarios. Water is used as a model material in calculations.

To demonstrate potential applications of the freezing model, freezing times are calculated for different experimental protocols. Two shelf ramp rates and different vials and fill volumes are considered. The heat transfer coefficient, which was determined experimentally for the SCHOTT 6R vial, is assumed to be similar to other vials used in the simulations. The results are presented in Fig. 2. Input parameters in calculations are presented in Table A1 (Appendix).

Figure 2 demonstrates that 1 h hold time would be sufficient for fill volumes of less than 1.5 mL (2R vial) and 5.5 mL (20R vial) at 1C/min ramp rate (fill depth 1 cm for both 2R and 20 R- table A1) which is in alignment with Tang and Pikal [1].

Note that freezing model data for high-fill volume products need to be used carefully since the model does not account for the time of ice propagation. Additional soak time (about 30 min) can be added to calculated values shown in Fig. 2.

The temperature of the fully frozen product is assumed to be -44.8° C rather than -45° C. Indeed, the product temperature is slowly approaching the final shelf temperature (-45° C) during the solid cooling stage in most cases. The final temperature change between -44.8° C and -45° C takes a significant amount of time and is not practical to be accounted for in real applications.

### Primary Drying

Since primary drying is usually the longest step and common risks, include collapse or meltback, this segment of the freeze-drying process has attracted the most effort. The critical first step in establishing primary drying conditions is selecting the target product temperature, commonly chosen as 2°C below the critical product temperature. Once the target product temperature is identified, shelf temperature and chamber pressure combinations are evaluated to establish a primary drying design space. An experimental approach for investigating the design space would require the manufacture of multiple batches [40] and therefore is very expensive. Computer modeling can reduce the cost associated with thoroughly investigating the design space. The section starts with a discussion of the selection of target product temperature, followed by a detailed mapping of the design space of primary drying using a mathematical heat/mass transfer model.

#### Target Product Temperature

While reducing primary drying time would require running the process at a higher temperature, there is an upper limit for a product temperature from a product perspective. The upper-temperature limit is defined by critical product temperature. This section discusses the identification of a target product temperature for two main types of formulations.

##### Amorphous Formulations

Tang and Pikal[1] wrote: “*The product temperature should always be several degrees below Tc in order to obtain a dry product with acceptable appearance*.” This is still the most common approach for selecting the target product temperature; indeed, exceeding the collapse temperature would lead to less-than-elegant cake and could compromise other product quality attributes such as reconstitution time, residual water content [41] and potency recovery [42]. The subject of primary drying above collapse temperature has been re-visited, with several publications providing evidence that collapse does not necessarily lead to potency losses [43]; this is consistent with a brief theoretical justification based on the relationships between the kinetics of protein unfolding (due to cold denaturation) and viscosity of the freeze-concentrated solution, as provided in [1]. Moreover, it was found that (micro)collapse could improve long-term stability and shelf life [44, 45]. In addition, the subject of what is acceptable in terms of visual appearance has also been discussed [46], to demonstrate that product quality could remain within specification even if the cake appearance is not pharmaceutically elegant.

Nevertheless, the recommendation by Tang and Pikal [1] represents a very reasonable approach for the majority of situations nowadays: “*a small safety margin (2°C) be used if freeze-drying time is long (e.g., more than two days), a large safety margin (5°C) be used if freeze-drying time is short (*< *10 h), and safety margin of 3°C be used if primary drying time is somewhere between 2 days and 10h.*”.

Recent work by M.Pikal group showed that the safety margin could be calculated for a given product using a combination of statistical approaches (accounting for the variability of process parameters, vial heat transfer coefficients, and cake resistances) and a steady-state model of primary drying [47]. This work suggests a decision-making framework for choosing a safety margin that optimizes the drying speed while maintaining an acceptable rejection rate.

While critical product temperature can be measured in many cases by FDM and/or DSC, it may be worthwhile to confirm the critical product temperature by performing freeze-drying cycle(s) with the product in vials at the target fill volume at the product temperature close to or/and exceeding the FDM/DSC-determined Tc. Such experiments may show that the product could tolerate product temperature higher than per FDM/DSC measurements. In this respect, optical coherence tomography, which has been applied for 3-dimensional imaging of pharmaceutical formulations in vials during freeze-drying [48], could be a very valuable tool for measuring critical product temperature in real-life conditions.

Spatial temperature heterogeneity within the product in a vial should be considered in experimental monitoring of the product temperature during freeze-drying. The temperature at the sublimation surface is lower than at the bottom, with larger temperature gradients observed at aggressive primary drying conditions and higher fill volumes [49–51]. Due to the higher temperature in edge vials, the degree of microcollapse in these vials during the aggressive cycle would be higher (with the corresponding low specific surface area) than in the center vials.

##### Partially Crystalline Formulations

As recommended by Tang and Pikal[1], the manufacturability of formulations with low collapse temperature can be improved by adding a crystalline bulking agent with high eutectic temperature, such as mannitol or glycine. In this case, primary drying could be performed well above the Tg’ of the freeze-concentrated solution without visual macroscopic collapse (see also [52]). For this approach to be successful, a sufficient amount of the crystalline phase is needed; in one case, the critical crystalline/amorphous ratio (glycine/raffinose in that case) to avoid macroscopic collapse was determined to be approx. 1.2 [53]_**.**_ The target product temperature can be selected based on the secondary solute + ice melting temperature. The Tsc (temperature of secondary crystallization/melt) is commonly above -5°C for both mannitol- and glycine-based formulations (exact value depends on the concentration and nature of other solutes present); therefore, the target product temperature could be conservatively selected as -10°C. However, performing primary drying at such high target product temperatures could overload an old freeze-dryer, as described in [1], who therefore recommended target product temperature not to exceed -15°C. Recent advances in freeze-dryer design could allow higher sublimation rates, so the target temperature could increase to at least -10°C. In such cases, considering that the product temperature is usually higher at the bottom of the product during primary drying, the target product temperature should be linked to the bottom product temperature rather than the temperature of the sublimation interface. Additional discussion of the overloading of a freeze-dryer and maximal sustainable sublimation rates is in the next section.

#### General Considerations of Primary Drying Conditions: Chamber Pressure and Shelf Temperature

In most cases, three main set points define primary drying: chamber pressure, shelf temperature, and primary drying time. The transition from freezing to the primary drying step is usually straightforward, with a vacuum applied at the end of the freezing segment, followed by heating the shelf to the pre-determined set-point, usually at the ramp rate of 0.5 or 1 °C/min. The heating power of commercial freeze-dryers can limit the ramp rate due to the high mass of shelves and product vials. Also, a fast heating rate may compromise the ability of the condenser to maintain the temperature below -40°C, and pressure control could be lost due to the high sublimation rate at the initial step. Chamber pressure during primary drying is almost universally maintained between 50 and 200 mTorr, with 100 to 150 mTorr representing a typical range for high Tg’ products. Such pressure range provides a compromise between the need to accelerate ice sublimation, which usually requires a lower pressure, and improves heat transfer from shelf to product, which benefits from higher pressure. Per Tang and Pikal [1], “*Pc should be well below the ice vapor pressure at the target product temperature to allow a high sublimation rate. The sublimation rate is proportional to pressure difference between the vapor pressure of ice and the partial pressure of water in the chamber (Pi), this difference being the driving force for ice sublimation. Pi is essentially the same as chamber pressure during primary drying. However, very low chamber pressure may cause problems, such as contamination of product with volatile stopper components or pump oil, and also produce larger heterogeneity in heat transfer, thereby giving larger product temperature heterogeneity between vials*”. Note that a distinction should be made between P_i_ (partial pressure of water in the chamber) and P_ice_ (pressure over sublimation surface). In calculations of sublimation rate, pressure difference P_ice_-P_c_ is used.

Also, note that a minimum sustainable pressure value depends on the equipment, sublimation rate, and chamber load, as discussed later in the section.

As a starting point, chamber pressure for primary drying can be selected using the following equation [1]:

1 $${P}_{c}=0.29\bullet {10}^{\left(0.019\bullet {T}_{P}\right)}$$

where *P*_c_ is chamber pressure (Torr) and *T*_p_ is product temperature (°C).

A common approach for the shelf temperature program during primary drying is maintaining the same temperature during the entire primary drying. However, in some (rare) cases, variable shelf temperature during primary drying can be beneficial to accelerate drying while keeping the product temperature below the critical value. Such an approach can be easily executed in laboratory-scale experiments but requires caution in a manufacturing environment to minimize errors in programming multiple primary drying steps.

Two parameters, chamber pressure and shelf temperature, define product temperature and duration of the primary drying for a given product and freeze-dryer. The product temperature at the sublimation interface is always lower than the shelf temperature during the sublimation stage, and the difference can sometimes reach more than 40°C.

As an efficient way to select the initial combination of chamber pressure and shelf temperature for a particular product and freeze-dryer, we recommend using a heat/mass transfer quasi-steady-state mathematical model as the first step. The basis for the model can be found in [54], with additional details (e.g., vials heat transfer coefficients) provided in [1]. More complicated (non-steady-state) mathematical models are also available [55]. Any mathematical heat/mass transfer model, which describes product temperature and water loss during primary drying, has two main inputs, i.e., the amount of heat supplied to the product from the environment and the heat extracted from the product by ice sublimation. For quasi-steady-state models, these inputs are provided by the vial heat transfer coefficient and the resistance of the product dry layer to water vapor flow. The authors’ experience is that the more straightforward quasi-steady-state approach has demonstrated appropriate accuracy in most cases studied, and this paper focuses on these models.

#### Description of Primary Drying Models

STca Lyo is the Excel-based steady-state primary drying model [56] in which heat and mass transfer over the sublimation surface is balanced using Solver. It uses vial/shelf heat transfer coefficients as a function of pressure (for both center and edge vial), the resistance of cake as a function of dry layer height, and minimum controllable pressure as a function of sublimation rate as input parameters for the calculation of product temperature profile and drying time for a particular dryer (listed in the database provided in this paper). It allows the estimation of conditions when the freeze-dryer cannot control pressure set point at a full load.

VMCa Lyo is a Python™ based steady-state primary drying model based on the same assumptions as those in the STca Lyo model. The model was designed to execute multiple scenarios that enable the creation of a design space for input parameters. This model uses “while loops” as its solver to increase or decrease the product temperature until it satisfies the conditions.

LyoPRONTO [38] is a lyophilization simulation and process optimization tool. The tool includes several instruments for predicting different stages of the lyophilization process: freezing and primary drying. It is also capable of design space generation, cycle optimization, product resistance, and heat transfer coefficient determination. The freezing calculator is based on a 0D lumped capacitance model, which can successfully predict the freezing process time and its steps. The primary drying calculator is based on a vial's 1-D quasi-steady-state heat-and-mass transfer. It is assumed that the central vial is representative of the entire batch, and convective heat transfer is neglected. The primary drying optimizer determines the optimal chamber pressure and shelf temperature at each time step to minimize the total drying time under the existing constraints.

#### Primary drying database

1. Inputs in primary drying model: dry layer resistance

**Fig. 3:**
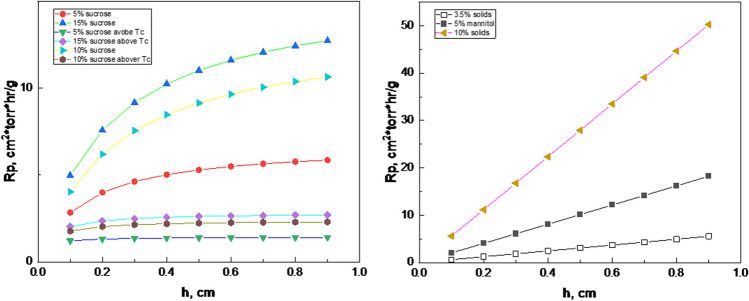
R_p_ for amorphous (left) and crystalline (right) formulations.

**Fig. 4:**
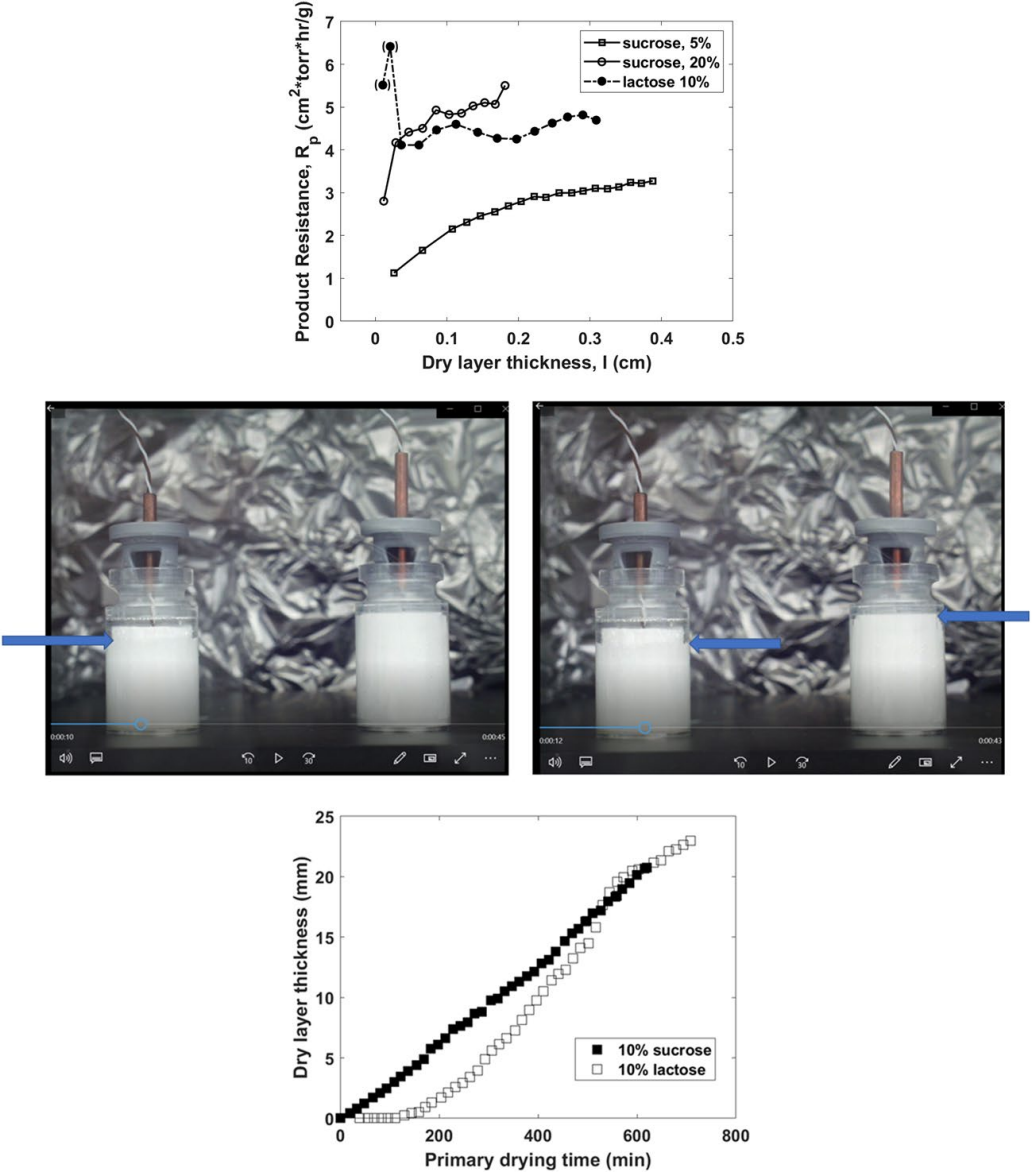
Top: Product dry layer resistance, R_p_, as measured by MTM in model solutions. Graph is prepared from data reported in [63], and the R_p_ data are from [64]. Middle: A snapshot of the video recording of the vials with 10% sucrose (left) and 10% lactose (right) during primary drying. The arrows show the sublimation front. Bottom: Graph is prepared from data reported in [63]. Dry layer thickness was measured visually during freeze-drying of 6 mL of sucrose and lactose solutions. Redrawn from [63]

**Fig. 5:**
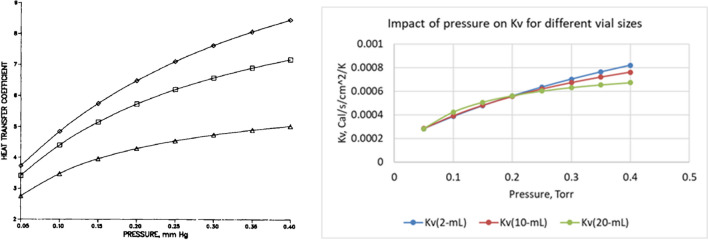
The vial heat transfer coefficient was calculated using the difference between shelf surface temperature and product temperature at the bottom of the vial (left panel, [54]) as opposed to K_v_, estimated as a difference between inlet temperature and product temperature at the bottom of vial (right panel).

Resistance of the dry product layer to the flow of water vapor depends primarily on the composition of the formulation and the dry layer thickness. The resistance can depend on the product history during freezing/annealing and primary drying, in particular, if the product experience change in the phase state (e.g., amorphous-to-crystalline transformation) or morphology (e.g., as a result of microcollapse) (Fig. 3). Dry layer resistance can be measured by MTM [57], PDM [58], TDLAS [59], micro X-ray tomography [60]**,** and calculated from a product temperature profile measured by a thermocouple [61]. In most cases, resistance increases as primary drying proceeds because of the increase in the dry layer thickness. A classical paper describes the measurement of cake resistances for different types of products [62], while examples of both common R_p_ patterns and unusual cases are provided below in Figs. 3, 4 and 5. Representative R_p_ patterns for typical amorphous formulations (model systems with different sucrose concentrations) and formulations with a crystalline bulking agent (mannitol) are shown in Fig. 3. These R_p_ patterns are used to assemble the primary drying database, as described later in the paper. For the sucrose formulations, the R_p_ increases at the beginning of the primary drying and tends to level off as the thickness of the dry layer increases due to likely microscopic collapse or difference in cake structure toward the bottom of the vial. The graph also shows two significant trends: higher resistance for formulations with higher solid content and a significant decrease in the resistance in cases when primary drying is performed to allow microcollapse. For crystalline formulations, the R_p_ tends to be higher than the amorphous of the same solid content, showing a near-linear increase with the dry layer thickness. It should be mentioned that the impact of the process on pore structure and cake resistance should be further investigated in a separate study correlating the pore structure of dry solids (for example, by micro CT) and resistance to mass flow.

The R_p_ for these formulations is described by an empirical equation [62]:

R_p_ = a + b*h/(1 + c*h), where h is a dry layer in cm, and a, b, and c are adjustable constants.

There are also cases when the R_p_ behavior deviates from the “standard” patterns. An example of such unusual behavior is presented in Fig. 4. for a model lactose system, a significantly higher resistance (i.e., a very low sublimation rate) was determined at the beginning of the primary drying [63]. Visual recording during primary drying showed a gradual progression of the sublimation front in a vial with 10% sucrose, while no sublimation was observed in the vial with lactose for the first 2 h of the primary drying (Fig. 4). This observation is consistent with the R_p_ measurements for 10% lactose performed using MTM. The very low sublimation rate for the lactose solution was attributed to a " skin " formation on the top of the frozen product. This would lead to a higher resistance to water vapor transfer, especially at the beginning of freeze-drying. However, it is still unclear why the skin would form in the lactose solution but not in the sucrose system.

2. Inputs in primary drying model: vial heat transfer coefficients

According to M.Pikal [54], a heat transfer coefficient is defined as the ratio of the area-normalized heat flow to the temperature difference between the heat source and heat sink. For the vials directly sitting on the shelf of the dryer (most common case), he defined the heat transfer coefficient, K_v_, as.

${K}_{v}=\frac{dq}{dt}\frac{1}{{A}_{out}}\frac{1}{({T}_{Sh.S-}{T}_{bottom})}$, where A_out_ is the cross-sectional area of the vial calculated from the vial outer diameter, T_Sh.S_ is the temperature of the shelf surface, and T_bottom_ is the temperature of the product at the bottom center of the vial.

As described by [54], the heat transfer coefficient (K_v_) is a function of pressure, heat radiation from the shelves (bottom and top), and direct contact between the vial and shelf. These three contributing factors are vial specific (depending, e.g., on the vial’s dimensions, wall thickness, and bottom concavity) and need to be measured in the experiment. Figure 5 (left panel) shows K_v_ = f(P) for W5816, K5816, and 5303 vials.

Measuring shelf surface temperature is difficult, especially for fully loaded dryers. As an alternative, the inlet temperature of heat transfer fluid is used to calculate K_v_ (Fig. 5, right panel). Note that K_v_, calculated from the temperature difference between inlet temperature and product temperature, is not only pressure or vial position-dependent (edge vials) but also varies from one freeze-dryer to another due to differences in shelf heat transfer coefficient. One can also notice a trend, with the heat transfer coefficient K_v_ typically decreasing with an increase in the size of vials, especially at high pressures.

According to [65] atypical radiation heat transfer is responsible for higher sublimation rates for vials located at the front and side of an array. This so-called “edge effect” could be as high as 50% [66] The edge effect (factor 1.5 used in simulations) is also an input parameter to the model.

3. Inputs in the primary drying model: minimum controllable pressure and maximum sublimation rate for a given freeze-dryer

**Fig. 6:**
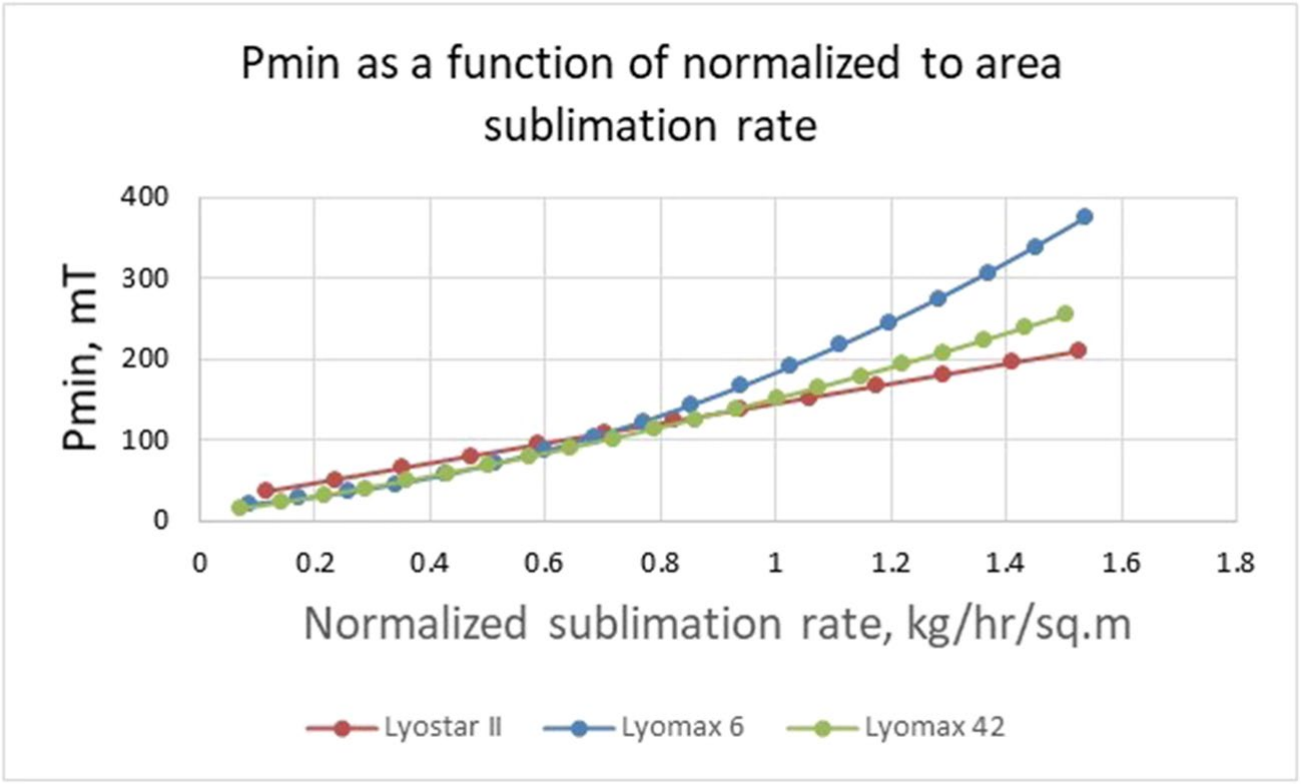
Minimum controllable pressure Pmin as a function of normalized (to total shelf area) sublimation rate for laboratory freeze-dryer Lyostar II (0.43 m^2^), pilot dryer Lyomax 6 (5.85 m^2^), and commercial-scale freeze-dryer Lyomax 42(41.85 m^2^).

Two critical characteristics of freeze-dryers are needed for the successful scale-up and transfer of lyophilization processes: minimum controllable pressure (or maximum sublimation rate at a given pressure) and maximum sublimation rate (typically due to condenser overload) for a particular dryer. The common understanding is that the dryer's performance could be limited by 1) the onset of choked flow, which limits the ability to control pressure, or by condenser capacity, where condenser temperature increases excessively. Due to geometric differences and limitations in the design laboratory, pilot and commercial dryers could respond differently to sublimation rates [67]. A total sublimation rate profile during simulation is calculated as a sublimation rate profile from the individual vial multiplied by the number of vials. Then, it is compared to the pressure the freeze-dryer could maintain at a particular sublimation rate (minimum controllable pressure, P_min_). Minimum controllable pressure could be obtained by either a sublimation test or by using computational fluid dynamics modeling (CFD) [68, 69]. An example of P_min_ as a function of sublimation rate for different freeze-dryers is shown in Fig. 6.

Note that normalized sublimation rate values, plotted in Fig. 6, showed up to 1.5 kg/hr/m^2^ while actual maximum sublimation rates values tested for these dryers were 0.8 kg/hr (Lyostar II), 5.45 kg/hr for Lyomax 6 and 50 kg/hr for Lyomax 42. In sublimation tests used during the characterization of these dryers, condenser temperature never exceeded -40°C, which we consider a critical point beyond which water vapor may not be able to condense fully, potentially resulting in a vacuum pump failure. Therefore, the true value of the maximum sublimation rate for these dryers would be when the condenser temperature is above -40°C. For most laboratory-scale dryers, the condenser temperature remains below -70C even when the flow is fully choked due to normally high refrigeration capacity. Therefore, the relationship between minimum controllable pressure and sublimation rate is typically linear (red line in Fig. 6). More details on this subject can be found in a recent paper [32]_**.**_

The normalized sublimation rate of 1.5 kg/hr/m2 could appear to be very high and nonrealistic. It corresponds to shelf temperature values above 40°C and pressures above 200 mT cycle conditions for the products with moderate cake resistance values (15 Torr*hr*cm^2^ at 1 cm for 10% solids, calculated as an example). Product temperatures, in this case, could exceed -10°C and stay just below the formulation's melting point during primary drying. While cycle conditions discussed above were not common in the past, in recent days and, especially in the future, one should expect more aggressive cycles to be implemented using bulking agents and freeze-drying well above Tg’. Most modern freeze-dryers can support high sublimation rates (up to 1.5 kg/hr/m^2^).

4. Verification of computational models

With R_p_ and K_v_ established and knowing the limitation of the freeze-dryer, primary drying conditions can be established using a computer model. Several primary drying-based models are listed in Table I. To compare the models, product temperature, sublimation rate, and primary drying time are calculated for multiple combinations of formulation/vial/fill volume/shelf T/chamber pressure; the complete dataset is provided in Supporting Information, Appendix (Tables A2-A4)1. Product temperature, primary drying time, and sublimation rate are similar between all three models As an example, product temperature at the bottom of the vial and primary drying time of the same product (5% mannitol in a 2-ml vial with a fill volume of 1.35 ml) and identical shelf temperature and chamber pressure (15 °C and 0.225 Torr, respectively) outputs for three models are provided in Table I. All three models provide comparable results. VMCa and LyoPRONTO product temperature traces for this example are also similar at every timepoint, with a maximum difference below 0.6 °C in two instances, within a range of 0.4 to 0.5 °C in 12 instances and below 0.3 °C for the rest of the data set (data not shown). The differences in the product temperature profiles between the VMCa and STCa data are negligible. The LyoPRONTO model shows a minor difference in the sublimation rate form the other 2 Lyocalculators. This can be explained because it uses a different type of solver, i.e., an optimizer package in Scipy, while the two other models use Excel-based and “while loops” solvers.

**Table I:** Comparison of primary drying computer models for Lyostar II, using 5% mannitol, 2-ml vial, fill of 1.35 mL, with Tsh = 15C and Pch = 225 mTorr

Computer model	Tpr (center), °C	Time (center), h	Tpr (edge)	Time (edge)
VMCa Lyo	-8.6	8.7	-5.7	7.0
LyoPRONTO Lyo	-8.8	8.6	-5.8	6.8
STCa Lyo	-8.6	8.8	-6.0	7.0

For any computer model to be valid, it is essential to confirm that computational results agree with the experiment. There are several publications in which different computer models of primary drying were compared with experiments. Specifically, a non-steady state PASSAGE model, which solves the heat and mass transfer equations in frozen and dried regions of a single vial by finite element formulation in 2-D axisymmetric space, has been verified for several freeze-dried products in glass vials [70]. Quasi-steady state approach, which commonly employs Excel-based calculations, has also been shown to accurately predict product temperature and primary drying time for several products [56].

**Fig. 7:**
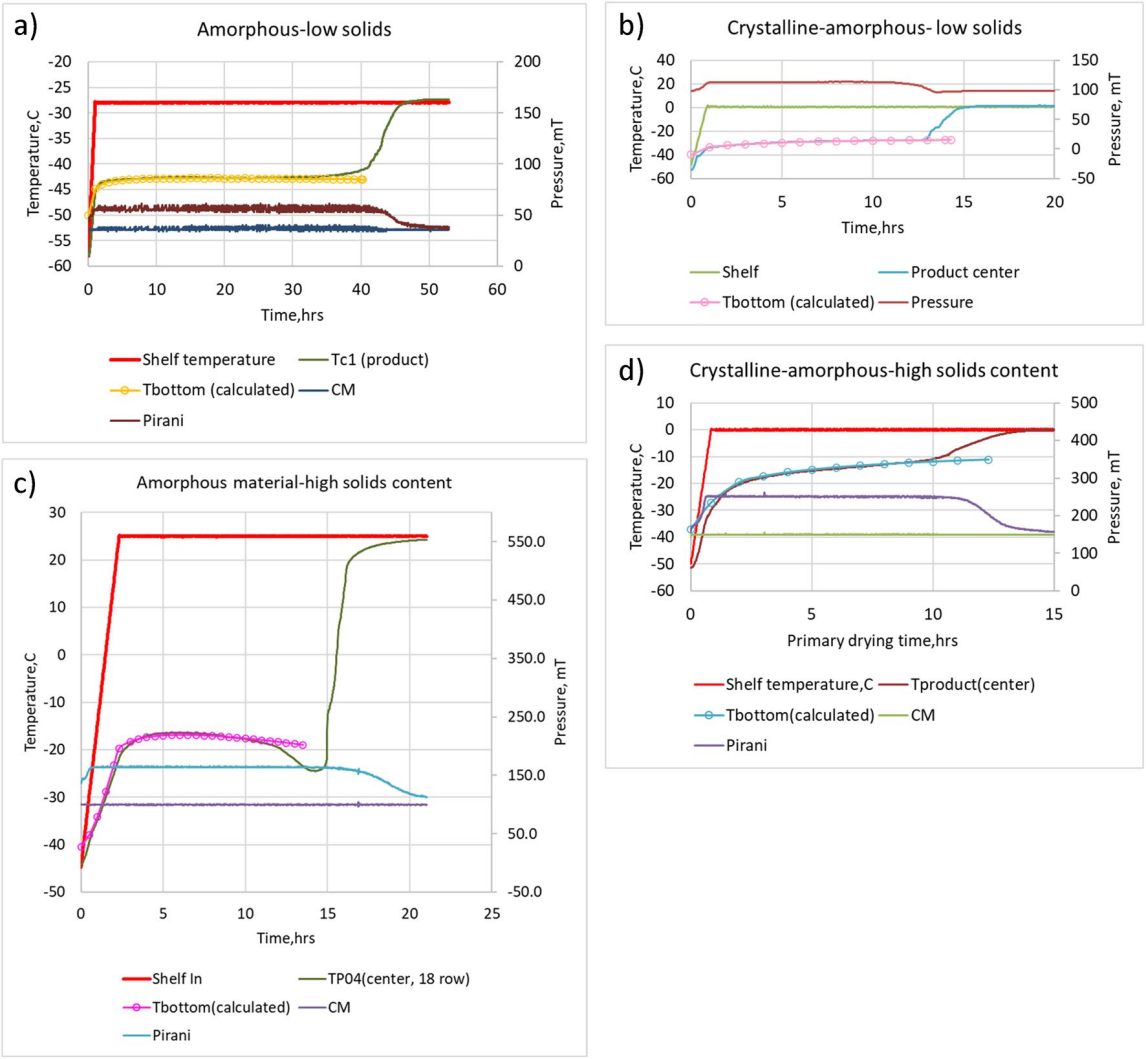
Comparison of experimental product temperature profiles during primary drying with the STCa model predictions: overlay of calculated and actual product temperatures for low solids content materials (amorphous-left upper figure (**a**) and crystalline-amorphous-right upper figure (**b**)) and high solids content materials (amorphous-left bottom figure (**c**) and crystalline-amorphous-right bottom figure (**d**)). Products and inputs for the model are provided in Table II.

In this study, the STCa Lyo model is compared with the experiment for four products (Table II), and the results are presented in Fig. 7 and Table III. The product temperature predicted by the model shows an excellent agreement with the experimental data, with the difference not exceeding 0.5° C. For the primary drying time, the most significant error (about 32%) was found in the prediction of drying time for high solids formulation (Table III). For the rest of the cases, the error in the prediction of drying time was less than 13%. Note that a comparison was made between the calculated and end point values estimated by the offset of Pirani, which is the most conservative approach to time prediction.

**Table II:** Input parameters for calculations of product temperature for materials in Fig. 11 and Table III

Material	Fill volume, mL	Solids content,%	Bulking agent	Annealing	Vial size*, mL	Input parameters	
						K_v_ *10^4^, Cal/cm^2/s	R_p_, Torr*hr*cm^2/g
Amorphous-low solids content	5.0	3.21	Dextran	No	20	A = 1.496 B = 22.276 C = 2.497	R0 = 0.396 R1 = 19.983 R2 = 17.379
Crystalline-amorphous-low solids content	4.0	3.41	Glycine	Yes	10P	A = 0 B = 70.000 C = 7.981	R0 = 0 R1 = 3.592 R2 = 0.155
Amorphous-high solids content	5.5	13.16	N/a	No	10	A = 1.430 B = 3.226 C = 2.745	R0 = 0 B = 19.427 C = 2.437
Crystalline-amorphous-high solids content	1.0	10.16	Mannitol	Yes	2	A = 0.532 B = 55.845 C = 5.854	R0 = 0 R1 = 55.838 R2 = 0

*All vials were manufactured by Schott, except 10P vial (10 mL Pinko)

**Table III:** Primary drying model vs. experiment: product temperature and primary drying duration, based on the results presented in Fig. 9

Product	Measurements	Tpr, °C		Primary Drying Time, h		
		Beginning of PD (set point is reached)	Middle of PD ( approximately 60% of PD)	Product T, experiment (offset)	Pirani, experiment offset	model
Amorphous-low solids content	Experiment	-46.10	-42.80	45.7	46.4	40.3
	STCa Lyo model	-44.90	-42.85			
Crystalline-amorphous-low solids content	Experiment	-34.57	-28.10	14.9	13.4	14.3
	STCa Lyo model	-33.21	-28.21			
Amorphous-high solids content	Experiment	-22.0	-17.0	16.7	19.8	13.5
	STCa Lyo model	-19.70	-17.22			
Crystalline-amorphous-high solids content	Experiment	-29.5	-13.70	13.0	13.2	12.3
	STCa Lyo model	-27.23	-13.34			

Overall, the STCa model, and by extension, two other models from Table I, are expected to provide an accurate estimate of the product temperature and a reasonable (within 30%, except for high solids case) prediction of the primary drying time.

All cycles were performed above Tg’ of formulations but below collapse (measured by freeze-drying microcopy) temperature.

Data of Table III show that the primary drying model (STCa) well predicts product temperature at the middle point of drying (approximately 60% from the beginning of the ramp) for four types of products. At the same time, the difference between the predicted value and actual measured product temperature is about 2°C in the beginning of PD, when the shelf temperature reaches the set point. Note that predicted drying time is always shorter than drying time measured either by product temperature or Pirani (in both cases using offset values). However, differences do generally not exceed 15% except for high solids content amorphous formulation.

5. Primary drying database for laboratory-scale LyoStar 2 freeze-dryer

To build the database, the following variables are included (Table IV):

i. product type, which defines dry layer resistance. The R_p_ could also depend on the cycle conditions, namely cooling/annealing conditions and the shelf temperature during primary drying. To account for the impact of the primary drying shelf temperature, two types of R_p_ for sucrose formulations are included: below Tc and above Tc.
ii. K_v_, which depends on vial size, chamber pressure, and the type of a freeze-dryer; in this database K_v_ for LyoStarII are used.
iii. Fill volumes.
iv. The number of vials is fixed to achieve the maximum load for LyoStar for each vial size, which is 1500 vials for 2 ml vials, 675 vials for 10 ml vials, and 420 vials for 20 mL vials.

**Table IV:** Ranges of input parameters for primary drying database

Input parameter	Range	Comment
Shelf temperature	-35 to 50°C	5°C step
Chamber pressure	20 to 450 mTorr	20 mTorr step at 20 to 100 mTorr, 25 mTorr step above 100
Vial type (nominal fill capacity)	2, 10, 20 mL	See Table A2 (Supporting Information) for HTC
Fill volume	0.67 to 20.9 mL	See Table A3
Formulation	5% mannitol; 5, 10, 20 wt% sucrose; Formulation-1 Formulation-2	See Table A4 for R_p_ Formulation-1 (3.5% solid): 2.5% glycine, 0.5% Sucrose, 0.4% protein, and a 0.1% buffer; Formulation-2 (10% solid): 4% mannitol, 1% Sucrose, and 5% protein

These parameters are used to calculate product temperature, primary drying time, and sublimation rates for the center and edge vials for 46,962 product/primary drying set-points combinations and presented as a database. Needless to say, it would be impossible to cover all combinations of input parameters. Nevertheless, the range of parameters selected here should cover most practical cases. A user could bracket a particular input parameter if a particular condition is not covered in the database. In some cases, two or more brackets could be required. Several examples of using the database are given later in the paper.

### Secondary Drying

The discussion in [1] is currently applicable in all aspects for secondary drying details. For example, a lower ramp rate for the amorphous product (within the range of 0.1 to 0.2 °C/min) can still be recommended to minimize the risk of collapse, while higher ramp rates (0.3 or 0.4°C/min) would be safe for crystalline or semi-crystalline products. Shelf temperature is the most important factor in the rate of secondary drying, while chamber pressure does not significantly impact either the rate of the extent of the secondary drying [71]. As a general rule of thumb for typical amorphous formulations, secondary drying can be completed in 3–6 h at shelf temperature 40 or 50°C, with water content below 0.5 wt% commonly achieved. While "the dryer = the better” is still the most common assumption, as reflected in residual water content specifications “no more than…” for all freeze-dried products, a possibility that a particular product may be more stable at some intermediate residual water content was mentioned in the original publication [1]. Additional studies describing examples and mechanistic assessments of relationships between water content and stability of amorphous materials have been provided more recently [72–75], confirming the earlier Tang and Pikal’s suggestion [1]**.**

**Fig. 8:**
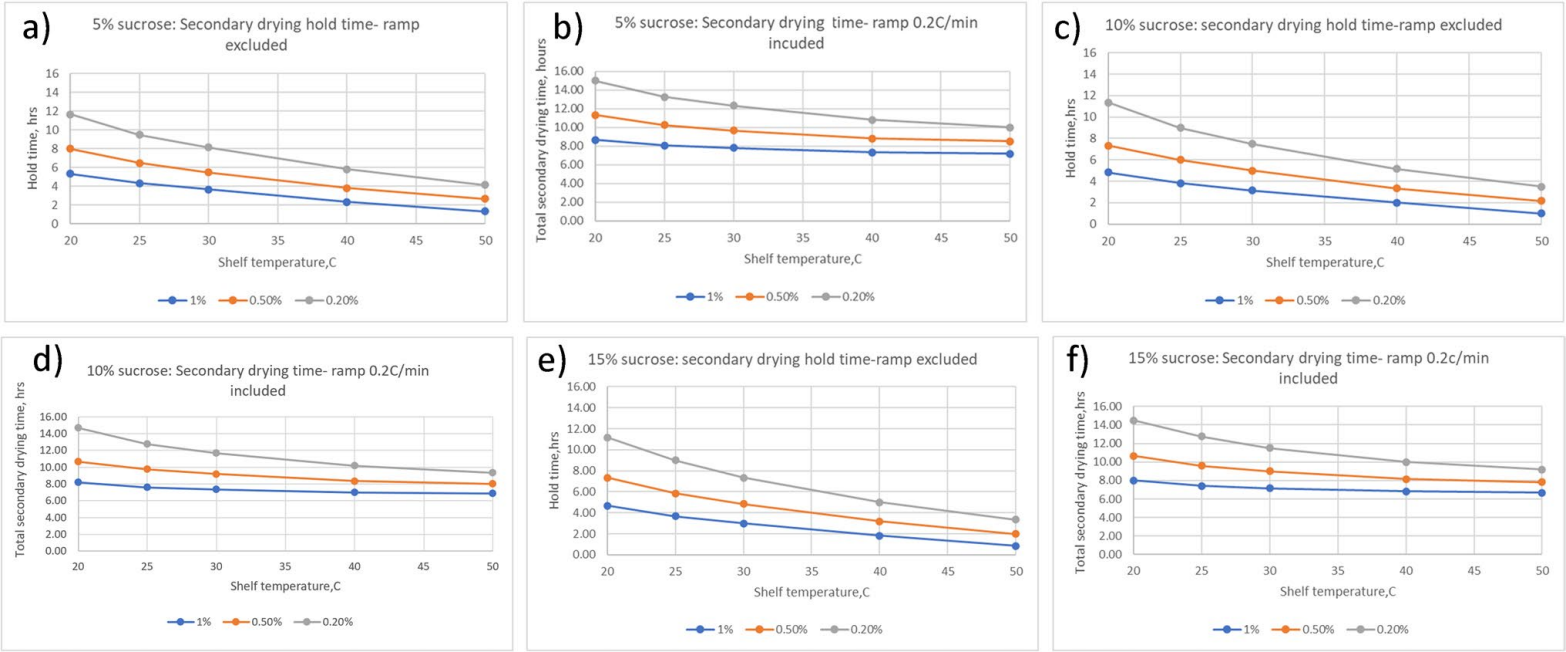
Secondary drying hold times for 5, 10, and 15% sucrose ((a), (c), and (e) panels respectively) as a function of shelf temperature to achieve residual moisture of 0.2% (grey), 0.5% (orange) and 1% (blue). The panels (b,d,f) represent the total secondary drying time (including ramp 0.2°C/min) for the same products. Calculations were made using Kg0 (pre-exponential factor [76],) values of 155.7 s^−1^, 142.94 s^−1^, 114.86 s^−1^ for 5%, 10%, and 15% sucrose, respectively, accounting for the change in specific surface area as a function of concentration [76].

An essential practical advancement is developing an Excel-based computational model to calculate secondary drying conditions [76]. An example of the model usage is provided in Fig. 8, in which secondary drying times for 5, 10, and 15% sucrose formulations are calculated at different shelf temperatures and target residual water contents. Only samples with controlled nucleation at -5°C were used in our calculations. For example, Fig. 8 shows for 5% sucrose that increases in the shelf temperature from 20 to 50°C would lead to a 3 × reduction in secondary drying time, although the duration would be relatively short even at the lower temperatures. Note that despite a longer ramp to higher secondary drying temperature, total secondary drying time decreases with an increase in shelf temperature during this drying step. The “2ndary drying calculator” can be used as an additional tool in an engineer/formulator toolbox in freeze-drying cycle prediction and optimization efforts.

**Fig. 9:**
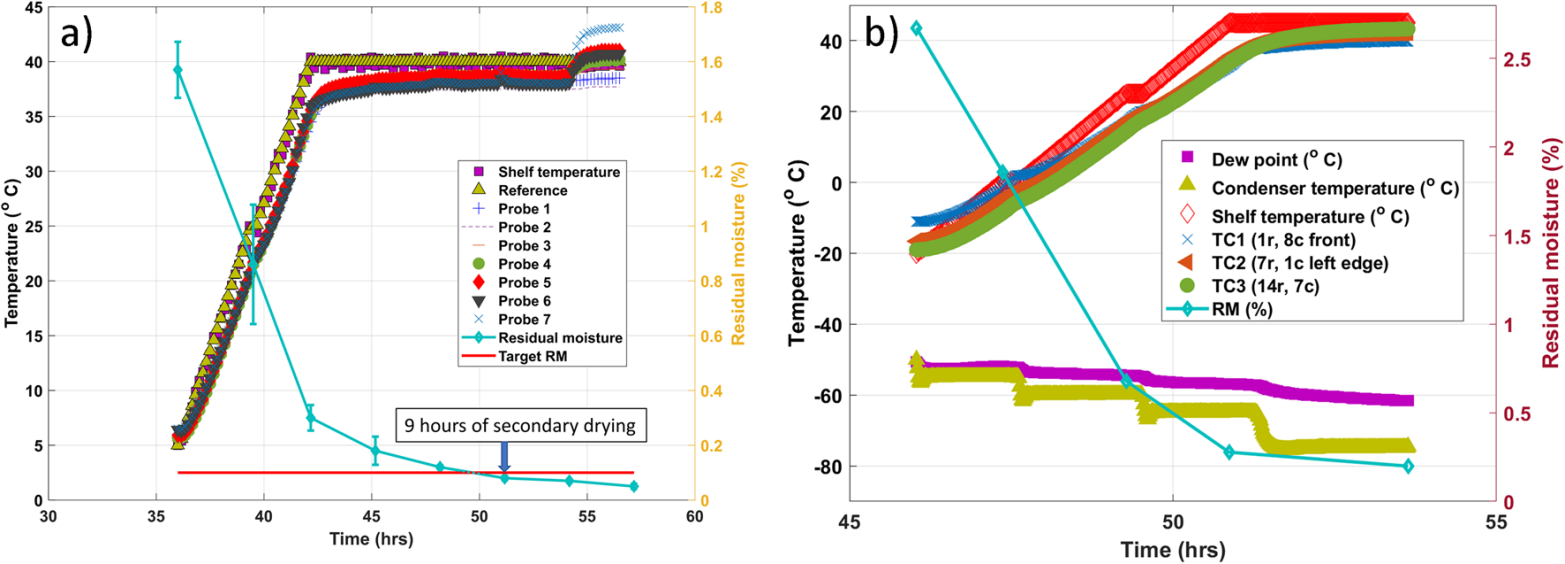
Kinetics of water removal for amorphous (left panel, the formulation contains 2% of small molecule and 4% stabilizer) and semi-crystalline (right panel, the formulation contains 4% mannitol, 1% stabilizer, and 1% protein) products. No annealing was used in the product shown in the left panel, while 2 h annealing step at -15°C was used in the semi-crystalline product.

Figure 9 represents the kinetics of water removal for amorphous and semi-crystalline products. Samples were stoppered at different secondary drying stages and analyzed for the Karl Fisher method after the completion of the cycle.

- Kinetics of water removal during secondary drying of product (small molecule)
- Effect of secondary drying conditions on moisture of crystalline/amorphous product

For amorphous products (about 6% solids), shown in Fig. 9, about 3 h of drying at 40°C are required to reach residual moisture below 0.2%. One can find from Fig. 8 that, for 5% sucrose, 5 h is needed to reach 0.2% residual water content at 40°C and 4 h at 45°C to achieve the same moisture. For semi-crystalline material (6% solids), 2.8 h at 45°C (high secondary drying temperature is needed to ensure removal of mannitol hemihydrate) is required to reach 0.2%. Given that this formulation's crystalline/amorphous content is 2:1 and assuming that all moisture resides in the amorphous phase, the actual moisture content in the sucrose/protein phase is likely about 0.6%. As can be found in Fig. 8, for 5% sucrose, it takes slightly above 3 h to reach 0.5% residual moisture at the shelf temperature of 45°C. Thus, in two examples shown in Fig. 9, the drying time to achieve target moisture was shorter, as was calculated for 5% sucrose. It can be assumed that the data in Fig. 8 are conservative estimates which can be initially applied to the design of a secondary drying step. To assess the impact of moisture on a particular product, samples at different points of the secondary drying step must be taken and subjected to long-term storage to reveal stability profiles as a function of residual water.

## Examples: Use of the primary drying database, and selection of freezing and secondary drying conditions

This paper considers examples of several hypothetical scenarios, although they do not cover all possible applications. The main starting points for selecting set-points for all three segments of the freeze-drying cycle include formulation description (crystalline/amorphous, total solid content), vials size, and fill volume. The selection of primary drying conditions starts with establishing the target product temperature for primary drying, Tt. For partially crystalline and amorphous formulations, the Tt is commonly selected as Tsc-2C and Tc-2C, respectively, although other approaches can also be used, as outlined below in example 2.3.

### Example 1

*Formulation with Crystalline Bulking Agent (4% mannitol + 1% amorphous content (stabilizer + active))*

*Inputs:* 5% mannitol, vial size 2 mL, fill volume of 1.35 ml.

A 20°C load temperature, 0.5°C/min cooling rate, and a freezing temperature of -45°C are recommended for the freezing cycle. Based on Fig. 2, 0.5 h hold time at -45 °C would be enough to reach product temperature below -44.8 °C. To crystallize mannitol, an annealing temperature of -10 °C^3^ for 3 h is suggested to ensure that crystallization can be efficiently done at the commercial scale. To keep product temperature below Tg’ even for the edge vials during vacuum initiation, re-freezing temperature can also be chosen about -45 °C^4^ for about 30 min. So, total duration of freezing step would be ((20 + 45)/0.5 + 30 + (45–10)/0.5 + 180 + (45–10)/0.5 + 30)/60 = 8.5 h. Cooling the condenser and pulling the vacuum to the set point typically takes about 1 h for a new dryer.

- *Primary drying*: using the drop-down menu in the Database, select “5% mannitol” formulation, vial size 2 mL, and fill volume of 1.35 ml.
- Next, the target product temperature is selected. The database has two product temperatures: the edge and center vial. The use of the edge vial is recommended as the safest approach. For example, 1, T_t_ = -5°C for the edge vial, based on the eutectic temperature for binary water/mannitol solutions of -2.2°C [77].
- Three product temperatures (edge) are selected as -5.01, -5.11, and -5.03C, which are closest to, but slightly lower than the T_t_. The former selection (T_p_ = -5.01C) results in an operational cycle, as shown in the last column of the database, while the cycle “will not work” for the last two product temperatures (-5.11 and -5.03C). The two cycles “will not work” because the recommended chamber pressure for these options would be lower than the minimal maintainable pressure for this freeze-dryer (Table V), assuming maximal vial load. The primary drying duration, in this case, is 8.6 h (cycle 1.1, Table V). Note that the “will not work” cases are based on the full freeze-dryer load; these conditions may work for a reduced load.

**Table V:** Process parameters for primary drying of 5% mannitol solution taken from the database. 10% edge effect (10% of edge vials received 50% more heat than center vials) was considered in calculations of the total sublimation rate for the fully loaded dryer (3shelvesx500 vials = 1500 of 2-mL vials)

Process Parameters	Primary drying cycle		
Case	1.1	1.2	1.3
Product T, °C (edge)	-5.01	-5.11	-5.03
PD time, h (center)	8.6	8.7	8.7
Chamber pressure, Torr	0.4	0.1	0.08
Shelf T, C	10	30	35
Sublimation rate, kg/hr	3.60E-01	3.12E-01	3.08E-01
P_min_, Torr (from Fig. 6)	0.127	0.113	0.111

### Secondary drying

Due to the possible formation of mannitol hemihydrate [78], a secondary drying temperature of 40 °C is recommended, provided that the product can withstand a short-term 40°C exposure. To reach moisture content below 0.2% (below 1% in the amorphous phase, Fig. 8) 2 h at 40 °C should be enough. Total secondary during time (assuming ramp rate to set point of 0.4 °C/min) would be ((40–10)/0.4 + 120)/60 = 3.25 h.

Total cycle time would be 8.5 + 1 + 11 + 3.25 = 23.74 ~ 24 h.

**Fig. 10:**
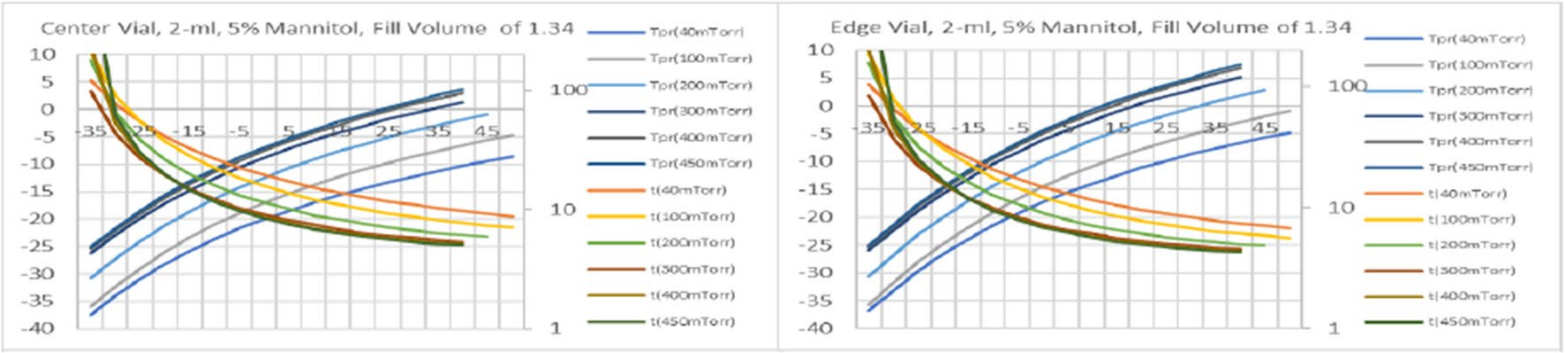
Design space for the case described in Table V. The design space is calculated using STCa Lyo model for the center (left panel) and edge vials (right panel). It is assumed that 10% of vials (edge vials) receive 1.5 more heat than center vials.

Using input parameters for the model (see appendix, tables A2-A4), a design space diagram showing the relationship between process parameters (shelf temperature and pressure) and product temperature and drying time could be built. An example of a design space diagram is shown in Fig. 10.

### *Example 2.*

A*n amorphous “sucrose-like” formulation, assuming that a SCHOTT* 2R vial is used and the target cake mass is 67.5 mg.”Sucrose-like” formulation would include any amorphous product with a collapse temperature close to -32C and an R_p_ profile similar to sucrose (Fig. 3, left panel). E.g., this could be a formulation with sucrose as the lyoprotector and low content of an active ingredient (e.g., protein). Three scenarios are considered for this example: a relatively low (5 wt%) total solid concentration of the bulk solution and a conservative cycle to maintain product temperature below the collapse temperature (2.1), a higher (10 wt%) total solid concentration of the bulk solution and a conservative cycle to maintain product temperature below the collapse temperature (2.2), and finally the 5 wt% total solid with more aggressive PD conditions in which the product temperature exceeds the macroscopic collapse temperature (2.3). Primary drying conditions are selected below for all 3 cases while freezing and secondary drying are included only for the first case.

- 2.1 Freeze-drying conditions for a model “5% sucrose” formulation with 1.35 ml fill, conservative cycle
- *Freezing:* 20 °C load temperature can be used. Let’s assume 0.5 °C/min rates during the freezing step and a freezing temperature of -45 °C. From Fig. 2, 0.5 h hold time at -45 °C would be enough to reach product temperature below -44.8 °C. Total freezing time would be ((20 + 45)/0.5 + 30)/60 = 2.67 h
- Cooling the condenser and pulling the vacuum to the set point typically takes about 1 h for a new dryer.
- *Primary drying*:
  Select the corresponding formulation from the menu as “5% Sucrose below Tc”. Select fill volume: 1.35 mL in this example. Select product temperature with a value close to but lower than the critical product temperature (Tc=-32C, and the corresponding Tt=-34C). Select three different product temperatures around the target product (Tt =-34C in this example), and check the last column to ensure it shows “operational”. In this particular case, 3 closest Tpr are -34.43, --35.28, and -35.32C. All three options give “Operational” conditions. The recommended shelf T/chamber pressure combinations are provided in Table VI. The 3d option, with the shelf T/chamber pressure -35C/150 mTorr, would result in a very long, and most likely unacceptable, primary drying time exceeding 500 hours. The two other options give a long but still realistic primary drying time estimate of approx. 60 hours. Option 2.1.2 provides the shortest cycle and pressure that can still be controlled.

**Table VI:** Primary drying process parameters for case 2.1

Process Parameters	Primary drying cycle		
Case	2.1.1	2.1.2	2.1.3
Product T, C (edge)	-34.43	-35.28	-35.32
PD time, h (center)	60.3	57.5	568.5
Chamber pressure, Torr	0.08	0.06	0.15
Shelf T, C	-30	-30	-35
Sublimation rate, kg/hr	0.0575	0.0603	0.00655
Pmin (from Fig. 10)	0.0388	0.0396	0.0241

- *Secondary drying*: Secondary drying of 30 °C may be chosen (as opposed to higher temperature) to minimize edge effect regarding moisture distribution across the shelf while still providing reasonable drying time. To reach target moisture of 0.5%, based on Figs. 7, 6 hours hold time would be enough if we assume the same desorption rate for this formulation as for 5% sucrose along. Assuming 0.2 °C/min ramp rate during secondary drying, total secondary drying time would be ((30 + 30)/0.2 + 360)/60 = 11 h.
- Total cycle time for 5% sucrose based formulation at about 1 cm cake height would be 2.67 + 1 + 78 + 11 = 92.67 ~ 93 h or about 4 days.

The calculated cycle with primary drying 2.1.2. option would still be long, with PD time approaching 58 h. There are at least two ways to reduce PD time without changing the composition of the dried formulation, i.e., either use higher concentration and lower fill volume or accept more aggressive PD conditions with the product temperature slightly above the Tc. These cases are described as Examples 2.2 and 2.3, respectively.

- 2.2 Primary drying conditions for a model formulation with 10% sucrose, 0.67 ml fill, conservative cycle.

Using the same approach as in Example 2.1 but with different inputs (10% sucrose, 0.67 ml fill, before Tc), the three Tpr (edge) closest to Tt = -34C are -34.14, -35.00, and -35.24C. The “-35.00” option “will not work because the corresponding chamber pressure would be too low (40 mTorr) to be controlled if the freeze-dryer is fully loaded. The latter option (Tpr = -35.24C) would correspond to the primary drying time (center vial) of > 300 h, which is practically unacceptable. Therefore, the recommended primary drying conditions would be Tsh = -30C and Pch = 60 mTorr, with the primary drying (center vial) of 30.8 h, corresponding to an almost 2 × reduction in the primary drying time compared with Example 2.1.

- 2.3. Primary drying conditions for a model formulation with 5% sucrose, 1.35 ml fill, aggressive cycle

For aggressive PD (5% sucrose after T_c_ option, see resistance coefficients in table A4), the target product temperature is selected slightly above the Tc = -32C. The 3 T_pr_ (edge) are -31.95, -31.78, and -31.99C. The last one (-31.99C) will not work, while the two other show “operational”, with recommended T_sh_/P_ch_ as -25C/175 mTorr and -15C/125 mTorr, respectively. The latter results in a shorter primary drying time of 16.6 h compared with 32.5 h for the -25C/175 mTorr. Therefore, -15C/125 mTorr can be recommended for primary drying. Such moderately aggressive PD conditions would reduce PD time by a factor of 3.5 at the same high fill volume of 1.34 ml (Table VII).

**Table VII:** Product temperature and primary drying time for Examples 2.2 and 2.3

Cycle output	5% Below Tc, 1.35 ml fill (3.1)		10% below Tc, 0.67 ml (3.2)		5% Above Tc, 1.35 ml fill (3.3)	
Tsh	-30		-30		-15	
Pch	80		60		125	
	Center	Edge	Center	Edge	Center	Edge
Product T, C	-35.4	-34.4	-35.2	-34.1	-33.1	-31.8
PD time, h	60.3	48.7	30.8	25.3	16.6	12.3

**Fig. 11:**
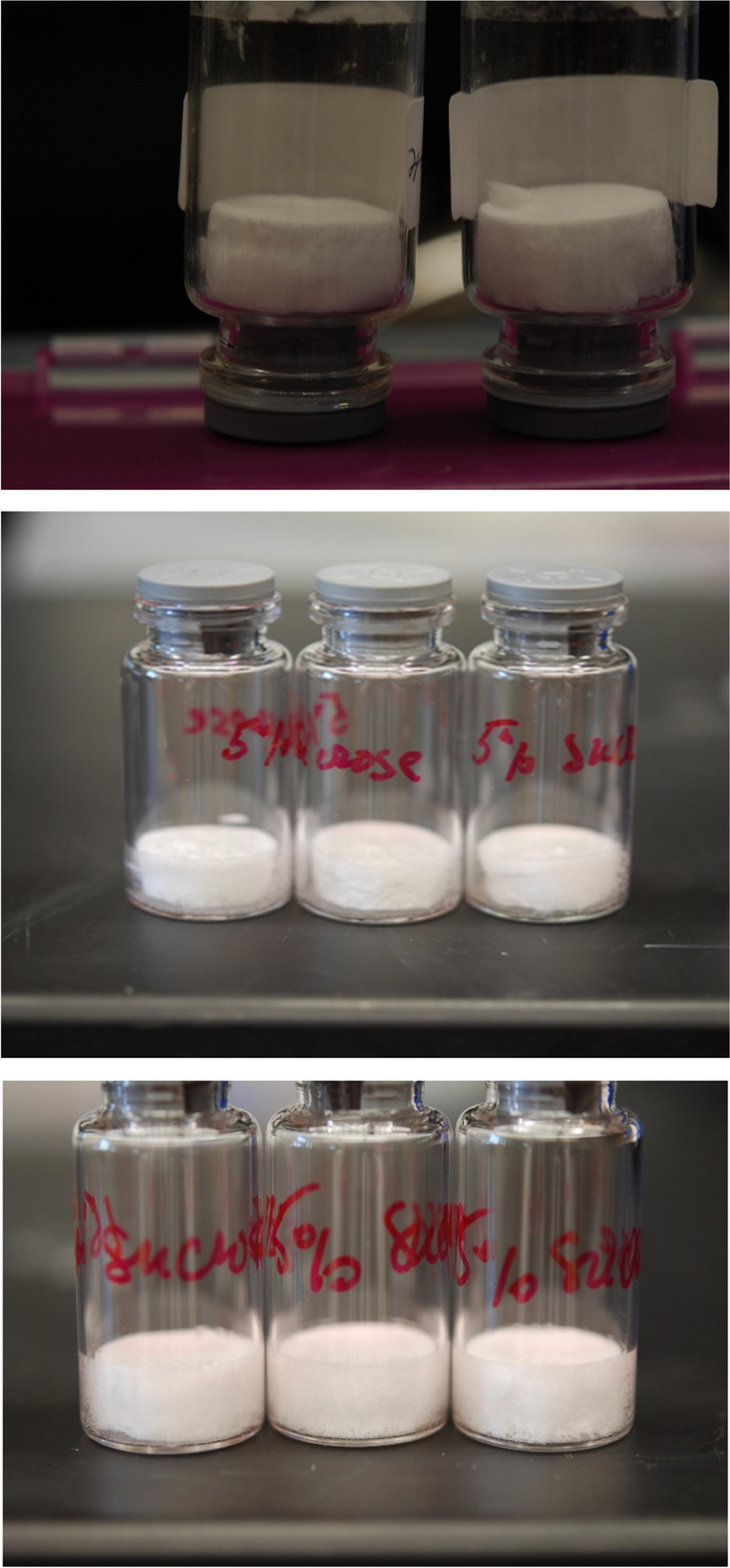
Cake appearance of 5% (top picture, left vial) and 15% (top picture, right vial) of sucrose lyophilized below microscopic collapse temperature (shelf temperature of -28°C and pressure of 50 mTorr resulting in product temperature below -36°C and 33°C for 5% and 15% of sucrose respectively). Lyophilized cakes are shown upside down to demonstrate no collapse at the bottom of cake. Middle and bottom pictures are 5% and 15% sucrose formulations, respectively, lyophilized at or slightly above microscopic collapse temperature (-32°C for 5% and -30°C for 15%) using shelf temperature of -10°C and pressure of 100 mTorr.

Note that performing the PD with product T slightly above the Tc would result in microcollapse, while the macroscopic collapse would still be avoided. The product appearance, in this case, would still be acceptable in most markets [46]. Below is an example of freeze-dried sucrose above and below microscopic collapse temperature (Fig. 11). Cake resistance calculated from this experiment was used in the generation of the database, coefficients for cake resistance are shown in table A4.

Note also that secondary drying for the “after Tc” cycle would be longer because of a lower specific surface area (because of microcollapse) and correspondingly lower water desorption rate. Usually, secondary drying time should be increased by approximately a factor of 2.

## Conclusion

Practical advice on the selection of freeze-drying process conditions from the seminal paper published in [1] are re-visited based on both the literature published since then and the recent experience of the authors of this manuscript. We found that most recommendations from the 2004 paper are as relevant today as they were 19 years ago, while some important advances are also noted. Relatively significant efforts have been devoted to developing methods to promote ice nucleation, with Praxair technology [10, 79], ice fog [3, 80], and vacuum-assisted freezing [11, 14] being the most pronounced, while several other methods including ultrasound [12] and spin-freezing [81] should also be mentioned. While some of these methods are being used on the laboratory scale, we are unaware of any such capabilities for commercial freeze-drying equipment. In addition, while controlled ice nucleation provided some benefits in some cases, there are also many examples of minimal differences between products made with controlled ice nucleation and conventional freezing. Overall, advice on freezing and annealing conditions [1] remain the same today. Computer modeling has become relatively common in primary drying, with both quasi-steady heat/mass transfer models being the most popular. Note that the primary drying modeling is based in a major part on Pikal’s studies [54]. Three such approaches are tested in this paper, and they deliver comparable results in terms of both product temperature, drying time, and sublimation rate predictions. One model is being used to assemble a primary drying database for various formulations, vial sizes, and fill volumes for multiple T_sh_/P_ch_ combinations. Several examples of the use of the model to calculate primary drying conditions are provided in the paper. For these examples, freeze-drying cycles are recommended covering all three freeze-drying segments. In addition, using the database to build design space for primary drying is included in one of the examples.

While huge with almost 47,000 combinations calculated, the primary drying database has two limitations. One is related to R_p_ vs. dry layer height patterns, with typical behavior for amorphous and crystalline formulations described by empirical questions. This approach leaves out products with “atypical” R_p_ patterns, and the current database might be less accurate in calculating primary drying conditions for such “atypical” formulations. However, based on the authors' collective experience, we argue that most real pharmaceutical formulations demonstrate “normal” R_p_ behavior. A more significant limitation is that the K_v_ values used in the database represent laboratory-scale LyoStar 2 freeze-dryers, and therefore the primary drying calculations are directly applicable to lab-scale freeze-drying only. Nevertheless, the approach described here can be used to build similar databases for the pilot- and commercial-scale freeze-dryers, provided that K_v_ values are determined.

To conclude, this paper may supplement the knowledge of scientists/formulators with significant freeze-drying experience and those new to this field. The database provides specific primary drying conditions for various formulations, vials, and fill volumes and examples of entire freeze-drying cycles to cover freezing/annealing, primary drying, and secondary drying conditions. For an advanced user, the database could represent a user-friendly tool to estimate the design space quickly and can also be used as a template to build a similar database to cover pilot- and commercial-size freeze-dryers.

We should mention that one of the important advances in lyophilization process development is an implementation of an instrument that allows direct measurement of sublimation rate (Tunable Diode Laser Absorption Spectroscopy- TDLAS ([59, 82, 83]). It may become the cornerstone of our approach to design space development for primary drying by enabling measurement of K_v_, R_p_, and equipment capability.

Also, the implementation of direct measurements of pore structure (micro CT) would enable a further tuning of heat transfer models by accurate estimation of resistance of porous cake.

## Supplementary Information

Below is the link to the electronic supplementary material.

Supplementary file1 (DOCX 18 kb)

Supplementary file2 (XLSX 9.61 mb)

## Data Availability

The authors declare that the data supporting the findings of this study are available within the paper and its supplementary information files. Data sets generated during the current study are also available from the corresponding author on reasonable request.

## Footnotes

1 In pharmaceutical freeze-drying literature, “eutectic” is commonly used to describe solute + water crystallization. This “eutectic” terminology, when applied to a typical pharmaceutical solution with several solutes present, is not correct from the physical chemistry perspectives. Eutectic is a non-variant (invariant) point on a temperature-composition (T-x) phase diagram of N-component system (where N is a total number of components including both solutes and solvent), which correspond to a co-existence of N + 1 phases (N is the number of components) for any condensed system, based on Gibbs phase rule. It means that for a binary system (e.g., mannitol + water), mannitol + water crystallization can be described as “eutectic” crystallization, while if the solution contains a third component (e.g., mannitol + sucrose + water), the “eutectic” term would require crystallization of all three components, i.e., mannitol, sucrose, and water. For such ternary system as water + mannitol + sucrose, the eutectic would mean co-existence of 4 phases (ice, crystalline mannitol, crystalline sucrose, and liquid). Such phase composition has not been observed experimentally even in such relatively simple systems as water + mannitol + sucrose. In essentially all real pharmaceutical solutions with N > 2, there is no eutectic crystallization during freeze-drying, at least eutectic in phys chem sense. We would recommend that a pharmaceutical scientist, who prefers to use “eutectic” to describe solute + water crystallization for aqueous solutions with 2 or more solutes, would add an appropriate disclaimer (e.g., [29]) to stress the difference from the “eutectic” terminology used in physical chemistry and other scientific fields.

2 There are also at least two other definitions of 'critical cooling rate'. The most common use of the 'critical cooling rate' terminology is in cryobiology, in which it describes primary water crystallization, i.e., freezing (water-to-ice transformation). This is different from the secondary solute + water crystallization, which takes place after the primary water crystallization. In a relatively dilute aqueous solution, 'critical cooling rate' for primary water crystallization would probably exceed thousands of degrees Celsius per minute, while 'critical cooling rate' for the secondary crystallization, as described in this paper, could be lower than a few degrees Celsius per minute. We would like to emphasize that we are using 'critical' in the physical–chemical sense, akin to 'critical micelle concentration', and not in the regulatory sense of a critical process parameter. This is a property of the formulation and in some cases, the cooling rate can be critical to quality attributes and needs to be evaluated. Another use of the same terminology of 'critical cooling rate' was introduced by [2], where it relates to different physical phenomena, i.e., to two patterns of freezing distinguishing between global supercooling and directional solidification.".

3 Our recommendation of -10 °C for mannitol annealing is based on understanding that the highest degree of crystallization and lowest amount of mannitol hemi-hydrate is based on practical understanding of such a behavior of mannitol. The temperatures above -10 C can induce the risk of potential melting in the edge vials. In addition, higher annealing temperatures could mitigate the formation of mannitol hemi-hydrate. Additionally, we advise utilizing elevated secondary drying temperatures, as it promotes a more comprehensive elimination of mannitol hemi-hydrate.

4 Our recommendation for re-freezing after annealing is made with caution and with the consideration that our proposed methods may need to cater to a variety of formulations, many of which may not be completely crystalline. In case of completely crystalline products, direct initiation of primary drying post-annealing is theoretically feasible. However, for biological products that are not entirely crystalline, ensuring that the product is fully solidified through re-freezing could be an essential step. Moreover, in some formulations with low collapse temperature, directly transitioning to primary drying from annealing might pose a risk. The rapid decrease in pressure when initiating vacuum could potentially induce degassing and/or (in extreme cases) boiling when the pressure drops below the water vapor pressure. This, in turn, can negatively impact visual appearance of the product, and also lead to a high mass flow rate during primary drying, potentially resulting in difficulties in maintaining the desired pressure setpoint. Therefore, a "safe" approach, which includes a re-freezing step after annealing is proposed. This will help to accommodate the range of products and formulations that may be subject to the described process. However, we acknowledge that for specific products and formulations where it's possible, avoiding the re-freezing step could save process time. Such an approach should indeed be validated through careful testing for each specific formulation.
