# Correction: Practical Advice on Scientific Design of Freeze-Drying Process: 2023 Update

**DOI:** 10.1007/s11095-024-03768-1

**Published:** 2024-10-07

**Authors:** Serguei Tchessalov, Vito Maglio, Petr Kazarin, Alina Alexeenko, Bakul Bhatnagar, Ekneet Sahni, Evgenyi Shalaev

**Affiliations:** 1 Pfizer Inc, Andover, MA USA; 2 Birck Nanotechnology Center, Purdue University, 1205 W State St., West Lafayette, IN 47907 USA; 3 Regeneron, Troy, NY USA; 4 Pharmaceutical Sciences, R&D, Abbvie, Irvine, CA USA

**Correction: Pharmaceutical Research (2023) 40:2433–2455**

10.1007/s11095-023-03607-9

The supplemental file has been replaced with the updated file.

## Supplementary Information

Below is the link to the electronic supplementary material.

Supplementary file1 (DOCX 15.8 KB)

Supplementary file2 (XLSX 9.61 MB)

