# Supplementary material for: Practical Advice on Scientific Design of Freeze-Drying Process: 2023 Update
Source: Pharm Res. 2023 Oct 2;40(10):2433–55. doi: 10.1007/s11095-023-03607-9 (PMC10661802; doi:10.1007/s11095-023-03607-9)
Supplement: Supplementary file 1 — Supplementary file1 (DOCX 18 kb) [file 11095_2023_3607_MOESM1_ESM.docx]

**Appendix: Supporting Information**

*Table A1: Input parameters for calculations of freezing time. Input parameters were taken from Schott brochure for 2R, 6R, 10R and 20R vials (https://www.schott.com/en-us/search/downloads?q=vials)*

| Vial | 2R | 6R | 10R | 20R | Frozen product thickness, cm |
| --- | --- | --- | --- | --- | --- |
| Din, cm | 1.4 | 2.0 | 2.2 | 2.76 |  |
| Dout, cm | 1.6 | 2.2 | 2.4 | 3.0 |  |
| Weight, g | 4.4 | 7.9 | 9.5 | 16.2 |  |
| Fill volume, mL | 0.71 | 1.44 | 1.74 | 2.75 | 0.5 |
|  | 1.41 | 2.88 | 3.49 | 5.49 | 1.0 |
|  | 2.12 | 4.33 | 5.23 | 8.24 | 1.5 |
|  |  | 5.77 | 6.98 | 10.98 | 2.0 |
|  |  |  | 8.72 | 13.73 | 2.5 |
|  |  |  | 10.47 | 16.48 | 3.0 |
|  |  |  |  | 19.22 | 3.5 |
|  |  |  |  | 21.97 | 4.0 |

Table A2: Coefficients for heat transfer coefficient for different vial sizes

| vial capacity, ml | 2 | 10 | 20 clear |
| --- | --- | --- | --- |
| a | 0.000167 | 0.000151 | 1.03E-13 |
| b | 0.002478 | 0.003006 | 0.008547 |
| c | 1.29419 | 2.4267 | 10.2034 |

Table A3: Different Fill Volumes, ml, that were used for primary drying database

|  | Vial nominal capacity |  |  |
| --- | --- | --- | --- |
| Cake heights, cm | 2-ml | 10-ml | 20-ml clear |
| 0.5 | 0.673932 | 1.595105 | 2.607318201 |
| 1 | 1.347864 | 3.190211 | 5.214636402 |
| 1.5 | 2.021796 | 4.785316 | 7.821954603 |
| 2 |  | 6.380422 | 10.4292728 |
| 2.5 |  | 7.975527 | 13.03659101 |
| 3 |  | 9.570633 | 15.64390921 |
| 3.5 |  |  | 18.25122741 |
| 4 |  |  | 20.85854561 |

Table A4: The cake resistant properties used for the databased

|  | **Crystalline Products** | | | **Below Collapse Temperature** | | | **Above Microcollapse Temperature** | | |
| --- | --- | --- | --- | --- | --- | --- | --- | --- | --- |
|  | **5% mannitol** | **3.5% solids** | **10% solids** | **5% sucrose** | **10% sucrose** | **15% sucrose** | **5% sucrose** | **10% sucrose** | **15% sucrose** |
| **a** | 0.000203 | 0 | 0 | 0.002893 | 0.003206 | 0.00316 | 4.27E-07 | 0.00013 | 0.0000153 |
| **b** | 20.23 | 6.156 | 55.838 | 48.827 | 57.689 | 72.6548 | 78.981 | 68.64008 | 72.597 |
| **c** | 0 | 0 | 0 | 7.2259 | 4.309 | 4.59248 | 55.127 | 28.75585 | 25.773 |

*Additional information, used in calculation of database:*

Equation S1 shows how density is calculated for these simulations. The equation is based on the density of sucrose. For this project, the total concentration was used to solve for the density of the vial. x is in terms of the total concentration and y is the density of the solution.

|  | $y=.0000177733*x^{2}+.003689*x+.9964$ | (S1) |
| --- | --- | --- |

The primary drying database is provided in supporting documents folder.
